# Development and validation of a targeted gene sequencing panel for application to disparate cancers

**DOI:** 10.1038/s41598-019-52000-3

**Published:** 2019-11-19

**Authors:** Mark J. McCabe, Marie-Emilie A. Gauthier, Chia-Ling Chan, Tanya J. Thompson, Sunita M.C. De Sousa, Clare Puttick, John P. Grady, Velimir Gayevskiy, Jiang Tao, Kevin Ying, Arcadi Cipponi, Niantao Deng, Alex Swarbrick, Melissa L. Thomas, Reginald V. Lord, Amber L. Johns, Maija Kohonen-Corish, Sandra A. O’Toole, Jonathan Clark, Simon A. Mueller, Ruta Gupta, Ann I. McCormack, Marcel E. Dinger, Mark J. Cowley, kConFab, Morteza Aghmesheh, David Amor, Lesley Andrews, Yoland Antill, Shane Armitage, Leanne Arnold, Rosemary Balleine, Patti Bastick, Jonathan Beesley, John Beilby, Ian Bennett, Anneke Blackburn, Michael Bogwitz, Leon Botes, Meagan Brennan, Melissa Brown, Michael Buckley, Matthew Burgess, Jo Burke, Phyllis Butow, Liz Caldon, David Callen, Ian Campbell, Deepa Chauhan, Manisha Chauhan, Georgia Chenevix-Trench, Alice Christian, Christine Clarke, Paul Cohen, Alison Colley, Ashley Crook, James Cui, Bronwyn Culling, Margaret Cummings, Sarah-Jane Dawson, Anna deFazio, Martin Delatycki, Rebecca Dickson, Joanne Dixon, Alexander Dobrovic, Tracy Dudding, Ted Edkins, Stacey Edwards, Maurice Eisenbruch, Gelareh Farshid, Andrew Fellows, Georgina Fenton, Michael Field, James Flanagan, Peter Fong, Laura Forrest, Stephen Fox, Juliet French, Michael Friedlander, Clara Gaff, Davi Gallego Ortega, Mike Gattas, Peter George, Graham Giles, Grantley Gill, Sian Greening, Eric Haan, Marion Harris, Stewart Hart, Nick Hayward, Louise Heiniger, John Hopper, Clare Hunt, Paul James, Mark Jenkins, Rick Kefford, Alexa Kidd, Judy Kirk, Jessica Koehler, James Kollias, Sunil Lakhani, Geoff Lindeman, Lara Lipton, Liz Lobb, Graham Mann, Deborah Marsh, Sue Ann McLachaln, Bettina Meiser, Roger Milne, Sophie Nightingale, Shona O’Connell, Sarah O’Sullivan, Nick Pachter, Briony Patterson, Kelly Phillips, Ellen Pieper, Edwina Rickard, Bridget Robinson, Mona Saleh, Elizabeth Salisbury, Joe Sambrook, Christobel Saunders, Jodi Saunus, Elizabeth Scott, Clare Scott, Rodney Scott, Adrienne Sexton, Andrew Shelling, Peter Simpson, Melissa Southey, Amanda Spurdle, Jessica Taylor, Heather Thorne, Alison Trainer, Kathy Tucker, Jane Visvader, Logan Walker, Rachael Williams, Ingrid Winship, Mary-Anne Young

**Affiliations:** 1 Kinghorn Centre for Clinical Genomics, Garvan Institute of Medical Research, Darlinghurst, NSW Australia; 2 Hormones and Cancer Group, Garvan Institute of Medical Research, Darlinghurst, NSW Australia; 3 St Vincent’s Clinical School, UNSW Australia, Sydney, NSW Australia; 4 The Sydney Head and Neck Cancer Institute, Chris O’Brien Lifehouse, Sydney, Australia; 5 Children’s Cancer Institute, Randwick, NSW Australia; 6 Endocrine and Metabolic Unit, Royal Adelaide Hospital, Adelaide, SA Australia; 7 Adult Genetics Unit, Royal Adelaide Hospital, Adelaide, SA Australia; 8 Department of Genetics and Molecular Pathology, Centre for Cancer Biology, an SA Pathology and University of South Australia alliance, Adelaide, SA Australia; 9 School of Medicine, University of Adelaide, Adelaide, SA Australia; 10 The Kinghorn Cancer Centre, Garvan Institute of Medical Research, Darlinghurst, NSW Australia; 11 St Vincent’s Centre for Applied Medical Research, Darlinghurst, NSW Australia; 12 Kathleen Cunningham Foundation Consortium for Research into Familial Breast Cancer (kConFab), Peter MacCallum Cancer Centre, Melbourne, VIC Australia; 13 Sir Peter MacCallum Department of Oncology, The University of Melbourne, Melbourne, VIC Australia; 14 Notre Dame University School of Medicine, Sydney, NSW Australia; 15 St George and Sutherland Clinical School, UNSW Australia, Sydney, NSW Australia; 16 Woolcock Institute of Medical Research, University of Sydney, Sydney, NSW Australia; 17 Northern Clinical School, The University of Sydney, Royal North Shore Hospital, St Leonards, NSW Australia; 18 Sydney Medical School, The University of Sydney, Camperdown,, NSW Australia; 19 Western Sydney University Medical School, Campbelltown, NSW Australia; 20 Australian Clinical Labs, Bella Vista, NSW Australia; 21 Department of Tissue Pathology and Diagnostic Oncology, Royal Prince Alfred Hospital, Camperdown, NSW Australia; 22 Central Clinical School, The University of Sydney, Sydney, NSW Australia; 23 Department for Oto-Rhino-Laryngology, Head and Neck Surgery, Inselspital, Bern University Hospital, University of Bern, Bern, Switzerland; 24 Department of Endocrinology, St Vincent’s Hospital, Darlinghurst, NSW Australia; 26 Illawarra Cancer Care Centre, Wollongong Hospital, Wollongong, NSW Australia; 27 Genetic Health Services Victoria, Royal Children’s Hospital, Melbourne, VIC Australia; 28 Hereditary Cancer Clinic, Prince of Wales Hospital, Randwick, NSW Australia; 29 Department of Haem and Medical Oncology, Peter MacCallum Cancer Centre, East Melbourne, VIC Australia; 30 Molecular Genetics Lab, Royal Brisbane and Women’s Hospital, Brisbane, QLD Australia; 31 Department of Translational Oncology, Westmead Hospital, Westmead, NSW Australia; 32 St George Hospital, Kogarah, NSW Australia; 33 Queensland Institute of Medical Research, Herston, QLD Australia; 34 Queen Elizabeth Medical Centre, Nedlands, WA Australia; 35 Silverton Place, Brisbane, QLD Australia; 36 John Curtin School of Medical Research, Australian National University, Canberra, ACT Australia; 37 Familial Cancer Centre, The Royal Melbourne Hospital, Parkville, VIC Australia; 38 NSW Breast Cancer Institute, Westmead, NSW Australia; 39 Department of Biochemistry, University of Queensland, St Lucia, QLD Australia; 40 Molecular and Cytogenetics Unit, Prince of Wales Hospital, Randwick, NSW Australia; 41 Clinical Genetics Service, Austin Health, Heidelberg, VIC Australia; 42 Royal Hobart Hospital, Hobart, TAS Australia; 43 Medical Psychology Unit, Royal Prince Alfred Hospital, Camperdown, NSW Australia; 44 Replication and Genome Stability Cancer Division, Garvan Institute of Medical Research, Darlinghurst, NSW Australia; 45 Dame Roma Mitchell Cancer Research Laboratories, University of Adelaide/Hanson Institute, Adelaide, SA Australia; 46 Peter MaCallum Cancer Centre, East Melbourne, VIC Australia; 47 School of Psychology, University of Sydney, Sydney, NSW Australia; 48 St Vincent’s Hospital Cancer Genetics Clinic, The Kinghorn Cancer Centre, Garvan Institute of Medical Research, Darlinghurst, NSW Australia; 49 Genetics Department, Central Region Genetics Service, Wellington Hospital, Wellington, New Zealand; 50 Westmead Institute for Cancer Research, University of Sydney, Westmead Hospital, Westmead, NSW Australia; 51 St John of God Subiaco Hospital, Subiaco, WA Australia; 52 Department of Clinical Genetics, Liverpool Health Service, Liverpool, NSW Australia; 53 Department of Clinical Genetics, Royal North Shore Hospital, St Leonards, NSW Australia; 54 Epidemiology and Preventive Medicine, Monash University, Prahran, VIC Australia; 55 Molecular and Clinical Genetics, Royal Prince Alfred Hospital, Camperdown, NSW Australia; 56 Department of Pathology, University of Queensland Medical School, Herston, QLD Australia; 57 Molecular Genetics Department, Cambridge University, Cambridge, United Kingdom; 58 Department of Gynaecological Oncology, Westmead Institute for Cancer Research, Westmead Hospital, Westmead, NSW Australia; 59 Associate Genetic Counsellor, Royal North Shore Hospital, St Leonards, NSW Australia; 60 Department of Pathology, Peter MacCallum Cancer Centre, East Melbourne, VIC Australia; 61 Hunter Genetics, Hunter Area Health Service, Waratah, NSW Australia; 62 Clinical Chemistry, Prince Margaret Hospital for Children, Perth, WA Australia; 63 Department of Biochemistry and Molecular Biology, University of Queensland, St Lucia, QLD Australia; 64 Department of Multicultural Health, University of Sydney, Sydney, NSW Australia; 65 Tissue Pathology, IMVS, Adelaide, SA Australia; 66 South West Family Cancer Clinic, Liverpool Hospital, Liverpool, NSW Australia; 67 Clinical Geneticist, Royal North Shore Hospital, St Leonards, NSW Australia; 68 Epigenetics Unit, Department of Surgery and Oncology, Imperial College London, London, United Kingdom; 69 Medical Oncology Department, Regional Cancer and Blood Services, Auckland City Hospital, Auckland, New Zealand; 70 Psychosocial Cancer Genetics Research Group, Parkville Familial Cancer Centre, Melbourne, VIC Australia; 71 School of Molecular and Microbial Sciences, University of Queensland, St Lucia, QLD Australia; 72 Department of Medical Oncology, Prince of Wales Hospital, Randwick, NSW Australia; 73 Victorian Clinical Genetics Service, Royal Melbourne Hospital, Parkville, VIC Australia; 74 Tumour Development Group, Garvan Institute of Medical Research, Darlinghurst, NSW Australia; 75 Queensland Clinical Genetic Service, Royal Children’s Hospital, Herston, QLD Australia; 76 Clinical Biochemistry Unit, Canterbury Health Labs, Christchurch, New Zealand; 77 Cancer Epidemiology Centre, Anti Cancer Council of Victoria, Carlton South, VIC Australia; 78 Department of Surgery, Royal Adelaide Hospital, Adelaide, SA Australia; 79 Department of Medical Genetics, Women’s and Children’s Hospital, North Adelaide, SA Australia; 80 Familial Cancer Clinic, Peter MacCallum Cancer Centre, East Melbourne, VIC Australia; 81 Breast and Ovarian Cancer Genetics, Monash Medical Centre, Bentleigh East, VIC Australia; 82 Medical Psychology Research Unit, The University of Sydney, Camperdown, NSW Australia; 83 Centre for M.E.G.A. Epidemiology, University of Melbourne, Carlton, VIC Australia; 84 Southern Health Familial Cancer Centre, Monash Medical Centre, Clayton, VIC Australia; 85 Clinical Geneticist, Genetic Health Services, Monash Medical Centre, Clayton, VIC Australia; 86 Director Medical Oncology, Westmead Hospital, Westmead, NSW Australia; 87 Familial Cancer Service, Department of Medicine, Westmead Hospital, Westmead, NSW Australia; 88 Breast Endocrine and Surgical Unit, Royal Adelaide Hospital, Adelaide, SA Australia; 89 University of Queensland Centre for Clinical Research, University of Queensland, The Royal Brisbane and Women’s Hospital, Herston, QLD Australia; 90 Breast Cancer Laboratory, Walter and Eliza Hall Institute, Parkville, VIC Australia; 91 Medcial Oncology and Clinical Haematology Unit, Western Hospital, Footscray, VIC Australia; 92 Westmead Institute for Cancer Research, Westmead Millennium Institute, Westmead, NSW Australia; 93 Kolling Institute of Medical Research, Royal North Shore Hospital, St Leonards, NSW Australia; 94 Department of Oncology, St Vincent’s Hospital, Fitzroy, VIC Australia; 95 Centro Nacional de Investigaciones Oncologicas, Madrid, Spain; 96 Western Health and Peter MacCallum Cancer Centre, East Melbourne, VIC Australia; 97 Genetic Services of Western Australia, Subiaco, WA Australia; 98 Familial Cancer and Clinical Genetics, Royal Melbourne Hospital, Parkville, VIC Australia; 99 Tasmanian Clinical Genetics Service, Royal Hobart Hospital, Hobart, TAS Australia; 100 Department of Medical Oncology, Peter MacCallum Cancer Centre, East Melbourne, VIC Australia; 101 Parkville Familial Cancer Centre and Genomic Medicine, Melbourne, VIC Australia; 102 Familial Cancer Centre, Westmead Hospital, Westmead, NSW Australia; 103 Oncology Service, Christchurch Hospital, Christchurch, New Zealand; 104 Centre for Genetic Education, Prince of Wales Hospital, Randwick, NSW Australia; 105 Anatomical Pathology, Prince of Wales Hospital, Randwick, NSW Australia; 106 Peter MacCallum Cancer Centre, East Melbourne, VIC Australia; 107 School of Surgery and Pathology, Queen Elizabeth Medical Centre, Nedlands, WA Australia; 108 Breast Pathology, University of Queensland Centre for Clinical Research, Royal Brisbane and Women’s Hospital, Herston, QLD Australia; 109 South View Clinic, Kogarah, NSW Australia; 110 Research Department, Walter and Eliza Hall Institute, Melbourne, VIC Australia; 111 Hunter Area Pathology Service, John Hunter Hospital, New Lambton Heights, NSW Australia; 112 Obstetrics and Gynaecology, University of Auckland, Auckland, New Zealand; 113 University of Queensland, Herston, QLD Australia; 114 Genetic Epidemiology Laboratory, Department of Pathology, University of Melbourne, Melbourne, VIC Australia; 115 Cancer Unit, Queensland Institute of Medical Research, Brisbane, QLD Australia; 116 Research Department, Peter MacCallum Cancer Centre, East Melbourne, VIC Australia; 117 University of NSW, Prince of Wales Hospital, Randwick, NSW Australia; 118 The Walter and Eliza Hall Institute of Medical Research, Parkville, VIC Australia; 119 Molecular Cancer Epidemiology Laboratory, Queensland Institute of Medical Research, Herston, QLD Australia; 120 Family Cancer Clinic, St Vincent’s Hospital, Darlinghurst, NSW Australia; 121 Department of Genetics, Royal Melbourne Hospital, Parkville, VIC Australia; 122 Genome.One, Garvan Institute of Medical Research, Darlinghurst, NSW Australia

**Keywords:** Cancer genomics, Cancer genomics, Next-generation sequencing

## Abstract

Next generation sequencing has revolutionised genomic studies of cancer, having facilitated the development of precision oncology treatments based on a tumour’s molecular profile. We aimed to develop a targeted gene sequencing panel for application to disparate cancer types with particular focus on tumours of the head and neck, plus test for utility in liquid biopsy. The final panel designed through Roche/Nimblegen combined 451 cancer-associated genes (2.01 Mb target region). 136 patient DNA samples were collected for performance and application testing. Panel sensitivity and precision were measured using well-characterised DNA controls (n = 47), and specificity by Sanger sequencing of the Aryl Hydrocarbon Receptor Interacting Protein (*AIP*) gene in 89 patients. Assessment of liquid biopsy application employed a pool of synthetic circulating tumour DNA (ctDNA). Library preparation and sequencing were conducted on Illumina-based platforms prior to analysis with our accredited (ISO15189) bioinformatics pipeline. We achieved a mean coverage of 395x, with sensitivity and specificity of >99% and precision of >97%. Liquid biopsy revealed detection to 1.25% variant allele frequency. Application to head and neck tumours/cancers resulted in detection of mutations aligned to published databases. In conclusion, we have developed an analytically-validated panel for application to cancers of disparate types with utility in liquid biopsy.

## Introduction

Next generation sequencing (NGS) has revolutionised our understanding of cancer. New taxonomies defined by molecular features rather than tissue of origin are emerging as powerful tools to better diagnose and treat cancers, and are becoming routine clinical practice in oncology and pathology^1,2^. The unprecedented success of gene- or genetic pathway-targeting therapies such as imatinib^3,4^, gefitinib^5,6^ and trastuzumab^7^ over a decade ago was arguably the driving force behind this re-classification of cancer, with 75.6% of United States-based oncologists now using NGS to guide treatment decisions^8^. While whole exome/genome sequencing remains largely cost prohibitive^9,10^, targeted gene panels have proliferated in mainstream clinical care due to their relative affordability and focused application; allowing for simplified data interpretation by omitting less-relevant genes and reducing the number of variants of unknown significance, and increased depth of coverage to improve detection sensitivity^9,11,12^. While to date, standardised validation criteria for diagnostic accreditation do not exist due to the lack of uniformity across the multitude of NGS platforms and within NGS processes^13^, stringent validation guidelines have been published to provide standardised frameworks for clinical genetic tests^11,14,15^. Transparency in the literature has promoted the detailing of targeted panels’ development and validation across a variety of diseases, and highlighting their sensitivity, specificity and precision^9,16–18^; factors viewed as paramount for enabling clinicians to make important clinical management and treatment decisions^19^.

In oncology, targeted panels have been developed for detecting hereditary cancer, monitoring somatic changes in progressive cancer, and importantly for elucidating the landscape of genetic aberrations that occur across multiple cancers, and using this information to identify novel therapeutics or repurpose existing ones^8,19–21^. In a landmark study in 2013, Frampton *et al*.^22^ identified clinically-actionable mutations in 76% of 2221 tumours studied; a 3-fold increase in drug-actionable detection as compared with contemporary diagnostic tests including Sanger sequencing, mass spectrometric genotyping, fluorescent *in situ* hybridisation and immunohistochemistry. As an example of therapeutic repurposing, the HER2-amplified breast cancer treatment trastuzumab (Herceptin) has been shown to be effective in advanced gastric cancer, and potentially HER2 amplified pancreatic ductal adenocarcinoma^23,24^.

Targeted gene panels are now also being produced for liquid biopsy and ctDNA sequencing; an emerging tool that holds promise to monitor for cancer initiation or relapse, tumour burden and evolution including the onset of treatment resistance^25–28^. Unlike standard surgical-core biopsies, liquid biopsies can be performed repeatedly, non-invasively, and are not spatio-temporally constrained, that is, results reflect all individual tumour mutations in real-time; thus informing the broadest range of therapeutic options available.

In a preliminary study, we recently applied a targeted gene sequencing panel to 44 patients with familial pituitary tumour syndromes (FPTS) and detected rare germline variants across the 8 analysed genes (*AIP*, *MEN1*, *CDKN1B*, *PRKAR1A*, *SDHA*, *SDHB*, *SDHC*, *SDHD*) in 11 patients^29^. Recognising the utility of this approach to poorly studied cancers, we hypothesised herein that further developing, expanding and validating our targeted gene panel would benefit patients with other cancers of the head and neck, and beyond. We therefore aimed to re-access our extended FPTS cohort, and expand our panel by adding hundreds of known cancer-associated genes, validate its sensitivity, precision and specificity against several well-characterised controls, and then apply it to two patient cohorts with oral and cutaneous squamous cell carcinoma of the head and neck (OSCC and cSCC respectively). We also aimed to determine our panel’s utility in liquid biopsy by targeting known mutations in genes at decreasing variant allele frequencies (VAFs), in a commercially available synthetic standard.

## Methods

### Patients

DNA samples were obtained from patients with the following conditions: FPTS [n = 38 (blood-derived) and 10 (tumour-derived)], sporadic pituitary tumours [n = 10 (blood-derived) and 6 (tumour-derived)], OSCC [n = 39 (tumour-derived)], cSCC [n = 26 (tumour-derived)], miscellaneous [n = 3 adrenocortical carcinoma (1 blood-derived and 2 tumour-derived), 4 pancreatic ductal carcinoma (3 blood-derived and 1 tumour-derived)]. Informed consent had been provided, and prior ethics approval obtained through St Vincent’s Hospital Sydney Human Research Ethics Committee, NSW, Australia (FPTS, sporadic pituitary tumours and adrenocortical carcinomas; protocol X13-0109), and Royal Prince Alfred Hospital, NSW, Australia (OSCC, cSCC and pancreatic ductal carcinomas; protocols X13-0417, LNR/13/RPAH/582 and HREC/11/RPAH/329). All experiments were performed in accordance with relevant guidelines and regulations.

### Development of the cancer gene sequencing panel PV1 and PV2

Two methods were used for deciding the genetic targets of our panels. Firstly, genes were selected from the following commercially available panels: TruSight Tumour 26 and TruSeq Amplicon Cancer Panels (Illumina), SureSeq Solid Tumour panel (Oxford Gene Technology), Foundation One Panel (Foundation Medicine), OncoCarta Panels Versions 1–3 (OncoCarta) and Haloplex Cancer Research Panel (Agilent). While each of these panels except Foundation One target specific exons or mutational hotspots, our design incorporated the entire coding region plus 10 bp of flanking intron of each gene included. Secondly, considering our panel was to expand upon our previously published FPTS data^29^, we searched for more pituitary tumour targets by conducting a literature search on PubMed (https://www.ncbi.nlm.nih.gov/pubmed/) for genes associated with aggressive pituitary tumours, familial pituitary tumours, and embryonic pituitary development. In total 312 cancer-associated genes were chosen for panel version 1 – PV1 (Table 1), with a target region of 0.97 Mb across 4,837 exons. The panel prototype was designed using the Roche/NimbleGen EZ Choice Library NimbleDesign platform [https://design.nimblegen.com/nimbledesign/#/ (cat #06266282001)].

**Table 1:** Genes targeted in Panel Version 1 and added to Panel Version 2 (bold).

*ABL1*	*BLM*	*CENPE*	***EPAS1***	*FLT4*	*IKZF1*	*MAP3K1*	*NFE2L2*	*PIK3R2*	*RYR1*	*SUFU*
***ACTN4***	*BMP4*	***CGA***	*EPCAM*	*FOXA1*	*IL7R*	***MAP3K20***	*NFKBIA*	*PMS2*	***S100A9***	***SUPV3L1***
*ADAMTS6*	*BMP8*	*CHD1*	*EPHA3*	*FOXL2*	*INHBA*	*MAPK2*	***NGN2***	***POMC***	*SDHA*	***TBXAS1***
***ADAMTS7***	***BMPR1A***	*CHD7*	*EPHA5*	***FSHB***	*IRF4*	***MAST4***	*NKX2-1*	*POU1F1*	***SDHAF2***	*TCF7L1*
***ADBRK2***	***BMPR2***	*CHEK1*	*EPHB1*	***GADD45B***	*IRS2*	***MAX***	*NKX3-1*	*PPP2R1A*	*SDHB*	TERT**
***AHR***	*BRAF*	*CHEK2*	***EPBH2***	***GADD45G***	*JAK1*	*MCC*	*NME1-NME2*	***PRB3***	*SHDC*	***TESC***
*AIP*	*BRCA1*	***CHRM3***	*ERBB2*	*GATA1*	*JAK2*	*MCL1*	*NOTCH1*	*PRDM1*	*SDHD*	*TET2*
*AKT1*	*BRCA2*	*CIC*	*ERBB3*	*GATA2*	*JAK3*	*MDM2*	*NOTCH2*	***PRDM2***	*SETD2*	***TFG***
*AKT2*	*BRIP1*	***CITED1***	*ERBB4*	*GATA3*	***JPH2***	*MDM4*	***NOTCH3***	***PRDM8***	*SF3B1*	*TGFBR2*
*AKT3*	*BTK*	***CLDN11***	*ERG*	*GID4*	*JRK*	*MED12*	*NPM1*	***PRDX2***	*SHH*	***TH***
*ALK*	***C2CD3***	*COX2*	*ESR1*	*GLI2*	*JUN*	*MEF2B*	***NR0B1***	***PRKAA2***	*SIRT1*	***TMEM43***
*AMER1*	***CACNA1H***	*CREBBP*	***ESR2***	*GNA11*	*KAT6A*	*MEN1*	***NR2C2***	*PRKAR1A*	*SIX3*	***TMEM127***
*ANOS1*	*CACNA1S*	*CRKL*	*EZH2*	*GNA13*	***KCNA5***	*MET*	***NR3C1***	*PRKDC*	*SIX6*	*TNFAIP3*
***AOX1***	***CAPN1***	*CRLF2*	*FAM46C*	*GNAQ*	*KDM5A*	***MGAM***	*NRAS*	***PRLR***	***SKI***	*TNFRSF14*
*APC*	*CARD11*	*CRMP1*	*FANCA*	*GNAS*	*KDM5C*	*MGMT*	***NTR3***	***PROCA1***	***SLIT2***	*TOP1*
*AR*	*C8FB*	*CSF1R*	*FANCC*	***GNRHR***	*KDM6A*	*MITF*	*NTKR1*	*PROP1*	*SMAD2*	*TOP2A*
*ARAF*	*CBL*	*CTCF*	*FANCD2*	***GPR101***	*KDR*	*MLH1*	*NTRK2*	*PTCH1*	*SMAD4*	*TP53*
*ARFRP1*	*CCAR2*	*CTNNA1*	*FANCE*	*GPR124*	*KEAP1*	*MLL1*	*NTRK3*	*PTEN*	*SMARCA4*	***TPM3***
*ARID1A*	*CCNB1*	*CTNNB1*	*FANCF*	***GREM1***	***KIAA0226***	*MLL2*	*NUP93*	*PTPN11*	*SMARCB1*	***TPM4***
*ARID2*	*CCND1*	***CXADR***	*FANCG*	***GRIN1***	***KIAA0363***	*MLL3*	***OCLN***	*PTTG*	*SMO*	***TRIOBP***
***ARMC5***	*CCND2*	***CYP19A1***	*FANCL*	*GRIN2A*	***KIF1B***	*MMP2*	***OGDH***	***PYCR1***	***SNAP25***	*TSC1*
*ASXL1*	*CCND3*	*CYP2D6*	***FANCM***	***GRIN2B***	*KIT*	*MMP9*	***OR51B4***	***RAB18***	*SNIP1*	*TSC2*
***ATAD2B***	*CCNE1*	***CYP21A2***	*FBXO4*	*GSK3B*	*KLHL6*	***MMP17***	***OSBP***	***RAB1A***	***SNX3***	***TSHB***
*ATM*	***CCR10***	*DAXX*	*FBXW7*	*HESX1*	*KRAS*	*MPL*	*OTX2*	*RAC1*	*SOCS1*	*TSHR*
***ATP6V0A1***	*CD79A*	*DBF4*	*FGF2*	*HGF*	***LATS2***	*MRE11A*	*PAK3*	*RAD50*	***SOCS2***	***UBR4***
***ATPAF2***	*CD79B*	*DCAMKL3*	*FGF3*	*HIF1α*	*LGALS3*	*MSH2*	*PALB2*	*RAD51*	***SON***	***UCP3***
*ATR*	***CDC2L2***	*DDR2*	*FGF4*	*HMGA1*	***LHB***	*MSH6*	***PALLD***	***RAD51C***	*SOS1*	*UGT1A1*
*ATRX*	*CDC73*	*DDX3×*	*FGF6*	*HMGA2*	*LHX2*	*MTOR*	***PARP10***	***RAD51D***	*SOX2*	***UGT2B7***
*AURKA*	*CDH1*	***DICER1***	*FGF8*	***HMGB1***	*LHX3*	*MUTYH*	*PAX5*	*RAF1*	*SOX3*	***USP8***
*AURKB*	***CDK3***	***DMD***	*FGF10*	*HNF1A*	*LHX4*	***MX2***	*PAX6*	*RARA*	*SOX9*	*VEGFA*
*AXL*	*CDK4*	*DNMT3A*	*FGF14*	***HNRPU***	***LOXL1***	*MYC*	*PAX7*	***RARS***	*SOX10*	*VHL*
*BAP1*	*CDK6*	***DOC1***	*FGF19*	***HOXB13***	*LRP1B*	*MYCL1*	*PBRM1*	***RASA4***	***SPANXN2***	*WIF1*
*BARD1*	*CDK8*	*DOT1L*	*FGF23*	***HPGD***	*LRP2*	*MYCN*	***PCDH11X***	*RB1*	*SPEN*	*WISP3*
***BAX***	*CDK12*	***DPCR1***	*FGFR1*	*HRAS*	***LRRC50***	*MYD88*	***PDGFD***	*RET*	*SPOP*	*WNT4*
***BBC3***	***CDKN1A***	***DSPP***	*FGFR2*	***HSP90AA1***	***LTBP4***	***MYO5A***	*PDGFRA*	***REXO4***	***SPTA1***	*WNT9A*
*BCL2*	*CDKN1B*	***DST***	*FGFR3*	*IDH1*	***MAG***	***NBN***	*PDGFRB*	*RICTOR*	*SRC*	*WT1*
*BCL2L2*	CDKN2A (p14ARF)*	*EGF*	*FGFR4*	*IDH2*	***MAN1A1***	*NCAM1*	*PDK1*	*RNF43*	***SRP9***	*XPO1*
*BCL6*	CDKN2A (p16)*	***EGFL7***	***FH***	*IGF1R*	***MAP1LC3B***	***NDRG4***	*PIK3CA*	*RPA1*	***SSR3***	*ZFHX3*
*BCL9*	*CDKN2B*	*EGFR*	***FLNA***	***IGFBP5***	*MAP2K1*	*NF1*	***PIK3CB***	*RPTOR*	*STAG2*	*ZNF217*
*BCOR*	*CDKN2C*	***EGLN1***	*FLT1*	***IGSF1***	*MAP2K2*	*NF2*	*PIK3CG*	*RUNX1*	*STAT4*	*ZNF703*
*BCORL1*	*CEBPA*	*EMSY*	*FLT3*	*IKBKE*	*MAP2K4*	***NFE2L1***	*PIK3R1*	***RXRG***	*STK11*	***ZNF717***

^*^Two protein coding isoforms were targeted of the same gene. **Including promoter region.

Panel version 2 (PV2) was a refined design leveraging off PV1 data to include more probes to improve read depth over poorly covered regions, plus incorporate the coding region (and 10 bp flanking intron) of 139 new genes (Table 1) associated with pituitary tumours and pituitary function; acquired through further PubMed literature search and clinical consultation. The target region of PV2 was 2.01 Mb, for a total of 6,929 target exons and 451 genes.

We used 80 and 111 DNA samples to measure the performance metrics for PV1 and PV2 respectively (see *Bioinformatics* below). These comprised 31 samples from the patient cohorts, 6 extracted DNA samples from 4 cancer cell lines (liposarcoma 94T778, breast cancer SK-BR-3, and melanoma SK-MEL-28 and A375), and 43 control samples for PV1; and 105 samples from the patient cohorts and 6 controls for PV2.

### Controls

Several controls were obtained to test the ability of PV1 and PV2 to detect well-characterised single nucleotide variants (SNVs), small insertions and deletions (indels) and copy number variants (CNVs): (1) 42 controls from breast cancer patients with known inherited mutations in 11 genes (Supplementary Fig. S1). We only retained controls that kConFab reviewed and classified/endorsed (n = 35) to test kit sensitivity; (2) an AcroMetrix Oncology Hotspot Control (Thermo Fisher Scientific, cat #969056), which consists of a pool of synthetic oligos against a normal genomic DNA background encompassing 521 somatic and 34 germline mutations in 53 genes (Supplementary Fig. S1); (3) a pair of DNA controls for testing our panel’s sensitivity to CNVs was derived from the well-characterised breast cancer cell lines SK-BR-3 and BT-474; (4) four controls from individuals NA12878, NA24385, NA24149 and NA21143 (Coriell Institute, NJ, USA), whose genomes have been deeply characterised using 12 technologies used to calibrate, benchmark and validate genomic tools for clinical practice worldwide^30^. The kConFab controls, the cell lines and the AcroMetrix Oncology Hotspot Control were tested on PV1.

Two repeat kConFab control samples and a repeat of the AcroMetrix Oncology Hotspot Control where expected mutations were missed in PV1 due to lack of coverage, and the Coriell samples were tested on PV2.

### Panel specificity testing

Routine clinical practice in St Vincent’s Hospital’s Department of Endocrinology for patients with suspected FPTS, includes Sanger sequencing of the *AIP* gene. Sanger sequencing is required for panel specificity validation, as it verifies true negatives, that is, it confirms that alleles labelled as wild type by next generation sequencing panels, are truly wild type and not mutated as previously defined^18^. We accessed the Sanger sequencing data for 89 FPTS patients who had been sequenced with our panels for this study, and the aforementioned preliminary study^29^.

### Liquid biopsy for ctDNA

To test for the applicability of our panel to liquid biopsy, we acquired synthetic oligos of analogous lengths to ctDNA, representing 9 known mutations across 6 cancer-associated genes (Seraseq Circulating Tumor DNA (ctDNA) Reference Material, SeraCare Life Sciences, MA, USA; cat #0710-0018). The mutated oligos were provided at different allelic frequencies (5%, 1.25%, 0.625%, 0.125% and 0%) in a matrix containing human proteins to closely resemble human plasma.

### NGS library preparation and sequencing

#### Genomic DNA

Genomic germline DNA was extracted from peripheral blood leukocytes using the QIAamp DNA Blood Midi Kit (Qiagen; cat #51183), or from fresh-frozen tumour samples using Qiagen’s DNeasy kit (cat #69504), through Garvan Molecular Genetics. DNA library preparation for all PV1 samples was conducted on 100 ng of input using the KAPA Hyper Library Preparation Kit (Roche, cat #KK8504), according to manufacturer’s instructions. For PV2, library preparation was conducted using the KAPA HyperPlus Kit (Roche, cat #KK8514) according to manufacturer’s instructions. KK8514 uses enzymatic digestion rather than sonication to fragment genomic DNA as per KK8504.

NGS was performed using Illumina’s HiSeq2500 for all samples in multiplexed pools of 17–24/lane.

#### ctDNA

Libraries were prepared using the QIAseq Ultralow Input Library Kit (Qiagen; cat #180495), according to manufacturer’s instructions. Oligos were captured with PV1 and sequenced on Illumina’s NextSeq500 platform in multiplexed pools.

### Bioinformatics

Our bioinformatics pipeline had been accredited for Medical Testing (ISO15189) by the National Association of Testing Authorities, Australia (NATA)/Royal College of Pathologists of Australasia (RCPA) in 2016. In brief, raw fastq files were transferred to the cloud-based genomic analysis platform DNAnexus (www.dnanexus.com). Bioinformatic read alignment and improvement, variant annotation, conversion, filtration and prioritisation were conducted as previously published^31–33^; the exception being for ctDNA where read duplications were not marked. Germline and somatic variants were called using the Genome Analysis Toolkit’s (GATK) HaplotypeCaller (v3.3) in GVCF mode^34^ and VarDictJava (v1.4.6)^35^ respectively. Copy number variants occurring across entire genes was determined using CNVkit (v0.9.1)^36^, and within genes using DECoN^37^.

Panel performance metrics including number of total reads, number of unique reads, mean coverage across regions captured by each panel version and targeted genes specifically (RefSeq coding exons), read enrichment and read duplication were assessed using the GATK’s CollectHsMetrics (Picard) and DepthofCoverage tools. By using controls with well-characterised SNVs, indels and CNVs, we were able to measure the sensitivity and/or precision of PV1 and PV2 as previously defined^18^. To measure precision recall and sensitivity for the four Coriell samples, we added a hard filter step recommended by GATK for small panels and then applied vcfeval in RTG Tools^38^.

### Statistics

Statistical analysis was performed using R (v3.6). Differences between groups were measured by ANOVA and identified using post-hoc Tukey’s HSD test. For the comparison between two groups, t-test was employed. p < 0.05 was used to determine if results were significantly different.

## Results and Discussion

### Performance evaluation

**Figure 1:**
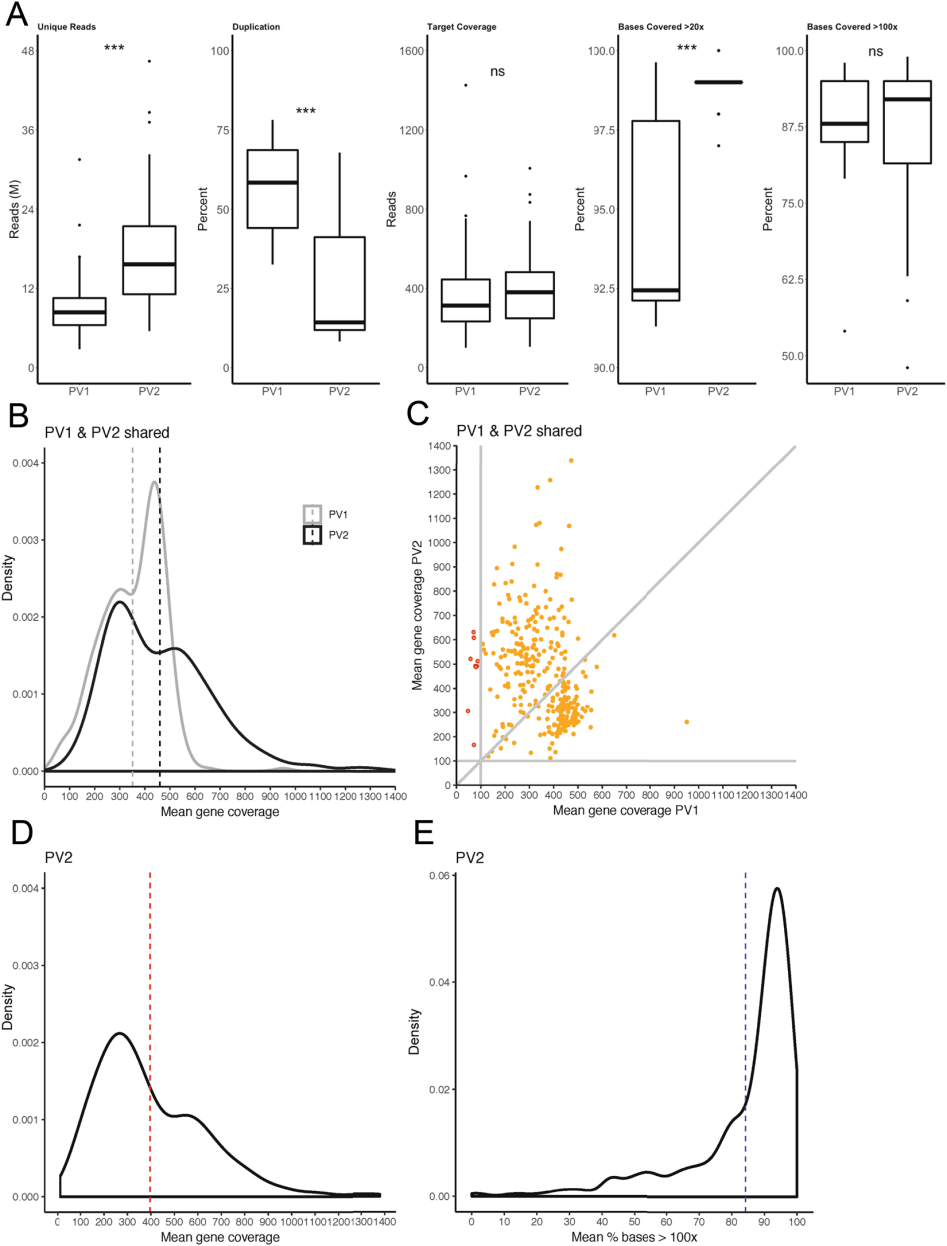
Performance metrics for PV1 and PV2. (**A**) Box plots presenting data comparisons between PV1 (n = 80 samples tested) and PV2 (n = 111 samples tested) for unique reads (in millions; M), percent duplication, target coverage and percent base coverage at >20x and >100x. (**B**) Mean coverage over coding regions of shared genes was compared between PV1 and PV2 after additional probes to target poorly-covered regions were added to the latter. (**C**) Changes to individual shared targets between PV1 and PV2 are also presented. (**D**) The final mean coverage for PV2 is presented following the addition of 139 new genes for a total of 451 targeted genes, as well as (**E**) the final percentage of targeted coding bases in PV2 with a percent mean coverage of >100x. Dashed lines in panels (**B–E**) represent mean coverage. ***p < 0.001, ns = not significant.

The mean total number of reads between PV1 and PV2 were similar at approximately 22.5 M (Supplementary Table S1), however the total number of unique reads for PV2 was nearly double that of PV1 (p < 0.001), consistent with the significantly lower PCR duplication rate in PV2 (p < 0.001) (Fig. 1A).

We achieved the vendor performance recommendations for both PV1 and PV2 designs, with a mean depth of coverage across regions captured by each panel version of >100x and a mean percent of captured bases covered at >20x across panel versions of ≥95% (Supplementary Table S1). While mean depth of coverage and percent bases with >100x coverage were not significantly different between PV1 and PV2 (Fig. 1A), greater variability was observed for PV1 for percent bases with >20x coverage, with a mean significantly lower than for PV2 (p < 0.001) (Fig. 1A). We then evaluated how well the designed probes covered the actual exons of targeted genes. As mentioned previously, exons identified with low coverage in PV1 were balanced with more probes in PV2. The result was an increase in the mean depth of coverage across the targeted genes shared between the panels, from 351x (PV1) to 460x (PV2) (Fig. 1B), and at individual gene level, the mean depth of coverage across samples was consistently >100x in PV2 but not in PV1 (Fig. 1C). If we take into consideration the full PV2 design, which includes an additional 139 genes, the global mean gene depth of coverage for this panel version was 395x (Fig. 1D) and the mean percent of targeted bases across genes with > 100x coverage was 84% (Fig. 1E).

Overall the performance of PV2 was an improvement upon PV1. Our panel far exceeds the typical 30x coverage recommended by providers of whole genome sequencing for germline analyses, and the 100x coverage recommended for somatic variant detection.

### Sensitivity assessment

#### kConFab

42 DNA samples from patients with mutations of various types in 11 genes were acquired from kConFab (Table 2, Supplementary Fig. S1), with the 35 that had been classified and reviewed proceeding through to library preparation and analysis. We detected 34 of the expected mutations (Table 2). The undetected variant, *BRCA1* c.135-1G > T, had been identified in the donor by two different clinical laboratories plus a research laboratory, and appeared to segregate with 12 other family members being affected. Our data however raises the potential of an annotation error, or potential sample swap, with just one read on the Integrative Genomics Viewer (IGV)^39^ exhibiting the mutation against a backdrop of ~1200 wild type reads of excellent mapping quality (Supplementary Fig. S2A). Two other potential annotation errors were observed among the detected variants (Table 2). Firstly, we detected *BRCA1* c.3193_3194dupG, p.Asp1065Glyfs*2, but it had been reported as c.3194_3195insG, p.Asp1065Glufs * 2 by clinical and research laboratories across numerous family members (Supplementary Fig. S2B). Secondly, we detected insertion *BRCA1* c.1879_1880insCC, resulting in a p.Val627Alafs*6 change (Table 2 and Supplementary Fig. S2C) instead of c.1881_1882_insCC, p.Ser628Profs*5 that was recorded by other laboratories. Next generation sequencing does have difficulties resolving indels in highly repetitive or homopolymer regions, as well as regions of high A-T and G-C content^15^, however the regions in Supplementary Fig. S2B,C do not appear to be problematic. As per the ‘undetected’ variant above, mapping quality for these two potential annotation errors was excellent, and excellent depth was achieved over both wild type and inserted alleles, providing confidence in our calls.

**Table 2:** Detection of kConFab variants.

Chr	Pos	Ref	Alt	Mut type	Gene	Detected CDS	Detected AA	Detection	VAF (%)
2	47702181	C	T	SNV	*MSH2*	c.1777C > T	p.Gln593Ter	D	48
2				DUP	*MSH2*	Exon 9-11 duplication		D	
2	215645969	GTT	G	DEL	*BARD1*	c.627_628delAA	p.Lys209Asnfs*4	D	51
9	97912356	G	A	SNV	*FANCC*	c.535C > T	p.Arg179Ter	D	53
9	98011506	TC	T	DEL	*FANCC*	c.67delG	p.Asp23Ilefs	D	49
10	89690810	G	T	SNV	*PTEN*	c.217G > T	p.Glu73Ter	D	56
10	89692818	TCA	CC	CV	*PTEN*	c.302_304delTCAinsCC	p.Ile101Thrfs*12	D	47
10	89692904	C	T	SNV	*PTEN*	c.388C > T	p.Arg130Ter	D	49
11	108165787	G	A	DEL	*ATM*	c.4909 + 1G > A	p.Met297_Ser301del	D	49
13	32913708	ATTAAGT	A	DEL	*BRCA2*	c.5217_5223delTTTAAGT	p.Tyr1739Terfs	D	53
13	32913778	T	A	SNV	*BRCA2*	c.5286T > A	p.Tyr1762Ter	D	45
13	32914174	C	G	SNV	*BRCA2*	c.5682C > G	p.Tyr1894Ter	D	50
13	32937635	AC	A	DEL	*BRCA2*	c.8297delC	p.Thr2766Asnfs*11	D	52
13	32954180	C	T	SNV	*BRCA2*	c.9154C > T	p.Arg305Trp	D	51
15	91328183	C	T	SNV	*BLM*	c.2695C > T	p.Arg899Ter	D	44
16	23632683	C	T	SNV	*PALB2*	c.3113G > A	p.Trp1038Ter	D*	45
16	23632683	C	T	SNV	*PALB2*	c.3113G > A	p.Trp1038Ter	D*	42
16	23634303	CA	CAA	INS	*PALB2*	c.2982dupT	p.Ala995Cysfs*16	D	43
16	23641239	CT	C	DEL	*PALB2*	c.2235delA	p.Lys745Lysfs * 19	D	51
16	23641528	CT	C	DEL	*PALB2*	c.1947_1948insA	p.Glu650Argfs * 13	D	44
17	7577539	G	A	SNV	*TP53*	c.742C > T	p.Arg248Trp	D	53
17	7578406	C	T	SNV	*TP53*	c.524G > A	p.Arg175His	D	39
17	7578552	G	T	SNV	*TP53*	c.378C > A	p.Tyr126Ter	D	45
17	7674945	G	A	SNV	*TP53*	c.586C > T	p.Arg196Ter	D	52
17				DUP	*TP53*	Exons 2–6 duplication		D	
17	41197774	A	T	SNV	*BRCA1*	c.5513T > A	p.Val1838Glu	D	49
17	41197784	G	A	SNV	*BRCA1*	c.5503C > T	p.Arg1835Ter	D	47
17	41243788	TAGAC	T	DEL	*BRCA1*	c.3756_3759delGTCT	p.Ser1253Argfs * 10	D	52
17	41244353	A	AC	INS	*BRCA1*	c.3193dupG	p.Asp1065Glyfs * 2	D^#^	52
17	41245666	T	TGG	INS	*BRCA1*	c.1879_1880insCC	p.Val627Alafs * 6	D^#^	45
17	41246260	C	CT	INS	*BRCA1*	c.1287dupA	p.Asp430Argfs * 6	D	45
17	41258551	C	A	SNV	*BRCA1*	c.135-1G > T	p.Phe46_Arg71del	ND^§^	
17				DUP	*BRCA1*	Exon 13 duplication		D^†^	
22	29095862	AG	A	DEL	*CHEK2*	c.1100delC		D	50
22	29121230	C	T	SNV	*CHEK2*	c.444 + 1G > A	p.Thr367Metfs	D^†^	41

Abbreviations: AA; Amino acid, Alt; Alternative, CDS; Coding sequence, Chr; Chromosome, CV; Complex variant, D; Detected, Del; Deletion, Dup; Duplication, INS; Insertion, Mut; Mutation, ND; Not detected, Pos; Position, Ref; Reference, SNV; Single nucleotide variant, VAF; Variant allele frequency. *The same variant was expected in two samples. ^#^Reported in kConFab depository as c.3194_3195insG, p.Asp1065Glufs * 2 and c.1881_1882insCC, p.Ser628Profs * 5 respectively. ^§^Visible as a single read against ~1200 wild type reads on Integrative Genomics Viewer. ^†^Detected on PV2 only.

**Figure 2:**
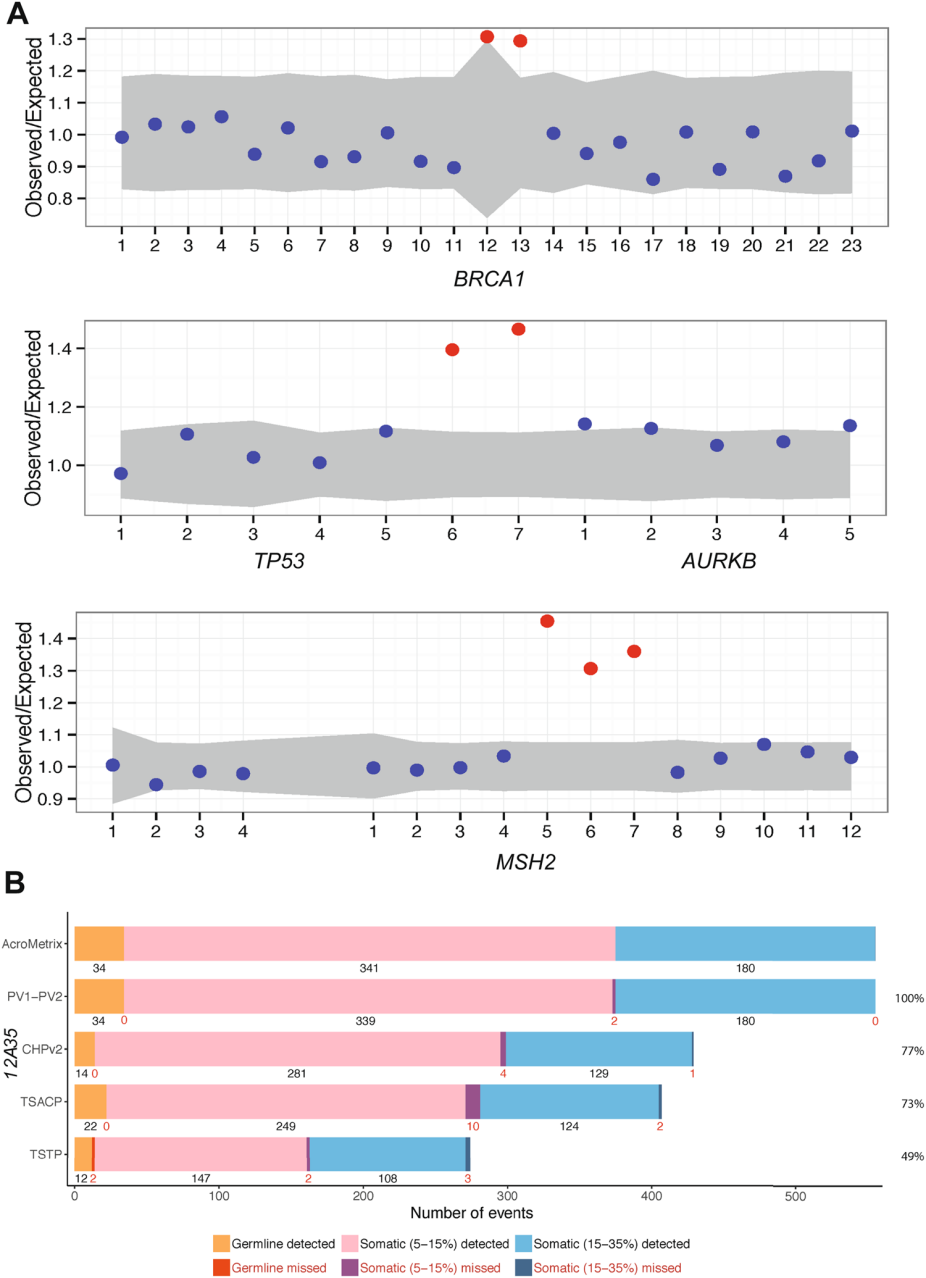
Intragenic copy number detection and comparison to commercial panels. (**A**) Control genomic DNA samples were acquired from kConFab for sensitivity testing. Three of these samples included known exon duplications in *BRCA1*, *TP53* and *MSH2*, which were assessed by the DeCON tool. Exons are numbered along the x-axis, and those of normal copy number are presented as blue dots. Amplifications are shown in red. The *TP53* and *AURKB* genes are on opposing DNA strands hence the presence of the latter and its exons in this Figure. A similar genetic-overlap is observed for *MSH2* to the left of the panel. (**B**) A commercially available pool of synthetic oligos against a normal genomic background was also obtained. Mutations were provided at variant allele frequencies (VAF) of 5–15% and 15–35%, or at germline frequencies. Presented are the number of detected and missed variants in our PV1 and PV2 panels relative to what was expected in AcroMetrix. This was compared to three other panels [AmpliSeq Cancer Hotspot Panel v2 (CHPv2), Illumina TruSeq Amplicon – Cancer Panel (TSCAP) and TruSight Tumor Panel 26 (TSTP)], the data for which were provided by the AcroMetrix manufacturer. Percent values on the right indicate the proportion of AcroMetrix variants actually targeted by the panels.

Two of the expected events, *BRCA1* exon 13 duplication (Fig. 2A) and *CHEK2* c.444 + 1G > A (Supplementary Fig. S3A), were only detected using PV2 after not being detected in PV1. This adds to the evidence of improved coverage in PV2 described above. In addition to the *BRCA1* duplication, two other expected multi-exonic duplications were successfully detected in *MSH2* and *TP53* using DECoN (Fig. 2A, Table 2).

#### Acrometrix oncology hotspot control (synthetic oligos)

PV1 detected 97% (n = 538) of the 555 expected mutations of various types across 53 genes (Supplementary Fig. S1, Supplementary Table S2) in the synthetic oligos. This included 98% of the somatic variants at 5–15% VAF, 97% of the somatic variants at 15–35% VAF, and 97% of germline variants. The addition to PV2 of new probes to cover poorly or non-targeted regions, greatly improved sensitivity, such that 553 (99.6%) of the expected mutations were detected (Figs. 2B and S3B). In comparison to the three other commercial sequencing panels presented in Fig. 2B, our panel had greater breadth, targeting 100% of the AcroMetrix oligo pool, and greater sensitivity, detecting proportionally more expected variants, with AmpliSeq Cancer Hotspot Panel v2 (CHPv2), Illumina’s TruSeq Amplicon – Cancer Panel (TSCAP) and TruSight Tumor Panel 26 (TSTP) detecting 98.8%, 97.1% and 97.4% of their respective targets.

With respect to the missed variants using PV2, both of which were expected at low VAFs (5–15%); the *HNF1A* c.872_873insC, p.G292fs*25 change, occurred in a highly repetitive region involving a poly-C tract, possibly creating alignment difficulties^15^ (Supplementary Fig. S3C). This is supported by the insertion being placed either side of the guanine nucleotide as shown in the top and bottom IGV panels of Supplementary Fig. S3C. Potentially compounding difficulties with this particular call, was the presence of *HNF1A* variant c.864G > C, p.G288G in the background genomic DNA. This variant was detected by both HaplotypeCaller and VarDict in both panel versions, with the former occasionally calling the correct insertion in PV1 at the same time. Consistent with this potential interference of accurate calling, was a large somatic indel (in addition to the substitution) called by VarDict in PV2 at position c.866_914. Thus in a real patient we may accurately identify c.872_873insC. We did detect all other expected indels and hence this is not an intrinsic issue with PV2. Our inability to detect *PIK3CA* c.1633G > A, p.E545K might reflect the threshold of detection of VarDict, since the variant was observed on IGV with 3.8% VAF (8/209 reads). However, this mutation occurs at a hotspot, with three other nearby mutations *in cis* all detected at the same VAF (Supplementary Fig. S3D).

Thus, while PV2 will not detect 100% of expected variants, its sensitivity of >99% is comparable to commercially available panels. Further, our ability to detect SNVs and indels at various VAFs confirms that our achieved coverage is sufficient for application to somatic cancer analyses. Nonetheless, we next investigated which variants of known clinical significance our panel would have difficulty detecting, using two well established databases; (i) ClinVar (https://www.ncbi.nlm.nih.gov/clinvar/), a catalogue of genomic variation and (ii) the Catalogue Of Somatic Mutations In Cancer (COSMIC; https://cancer.sanger.ac.uk/cosmic). Based on the literature, 20–30x coverage is sufficient for the detection of germline variants^15,40^. Assessment of our targeted regions which recorded mean depths of coverage of <20x, revealed that five targeted mutations of clinical significance in the ClinVar database, would be poorly covered and may not be identified (Supplementary Table S3). These five variants all occurred across three alleles within the same targeted region of the *LHB* gene. With respect to detecting somatic variants, 100x coverage is sufficient for detecting 80% of expected variants in tumour tissue of at least 55% purity^40,41^. Using 100x as a cut off, 158 targeted mutations encompassing 137 alleles in 23 genes in the COSMIC database may be missed in tumours of lower purity (Supplementary Table S3). These somatic alleles fall under 30 targeted regions of our panel, which when added to the poorly covered region identified in the ClinVar comparison, amounts to <0.5% of the total targeted region of our panel. Improvements could be made in future iterations of our panel design to compensate for these very few insufficiencies, or greater amounts of highly pure input DNA could be used at initial library preparations when searching for variants in these regions using PV2.

#### Control cell lines

**Figure 3:**
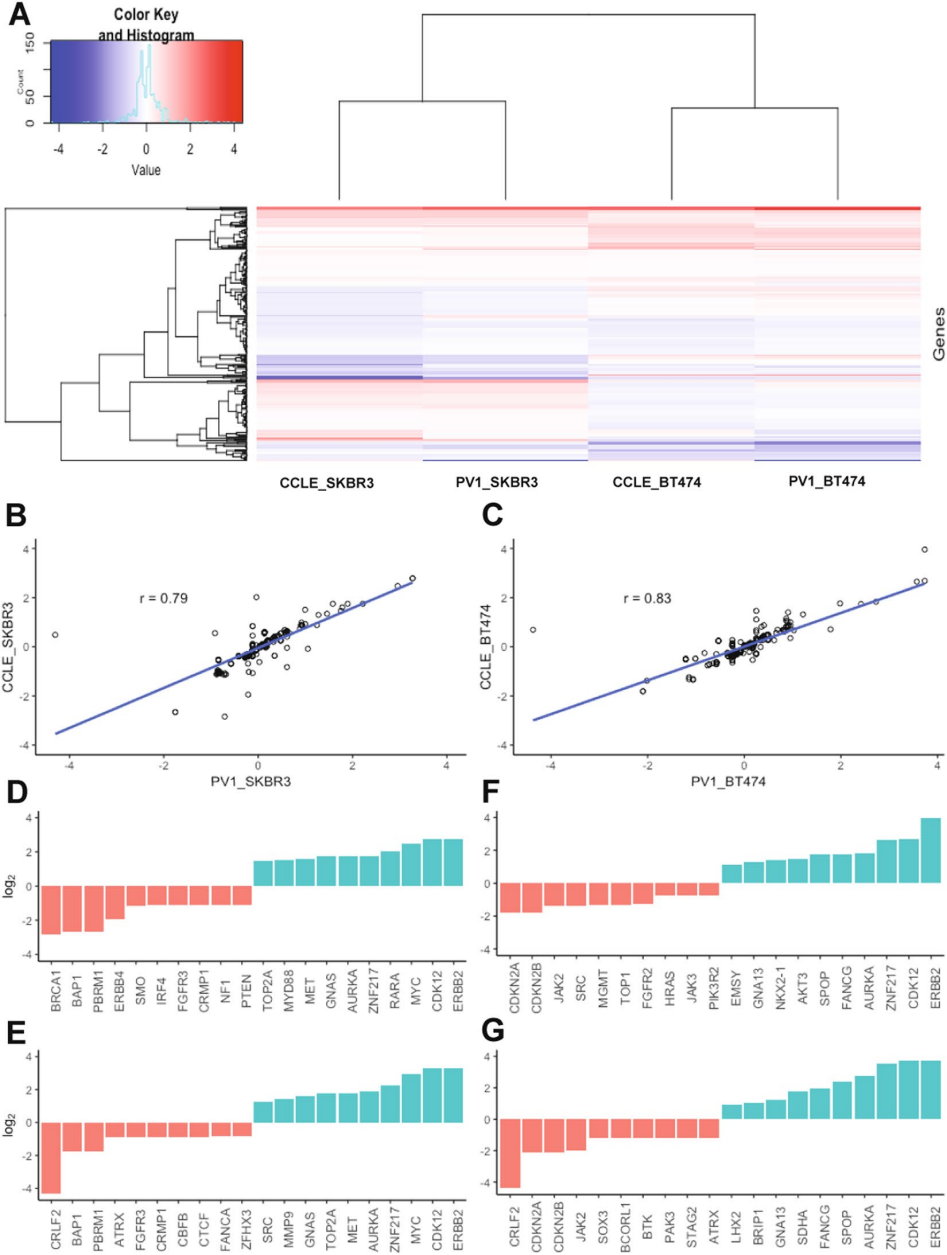
Copy number evaluation in breast cancer cell lines. (**A**) Heat map representation of ~300 targeted genes in the SK-BR-3 and BT-474 breast cancer cells lines. Data was extracted from PV1-captured DNA and processed through CNVkit and compared to those expected according to the Broad Institute’s Cancer Cell Line Encyclopedia (CCLE). Copy number deletions are represented in blue and as copy numbers approach diploidy colours become white. Amplification in copy number is represented in red. (**B**) Regression analysis was carried out using the correlation coefficient to quantify the strength of relationship between PV1 called variants in SK-BR-3 and (**C**) BT-474, and those expected according to CCLE. An r-value of >0.7 is considered a strong, positive relationship. (**D–G**) Ten lowest (red) and ten largest CNV (blue) changes as detected by PV1 in SK-BR-3 (**D**) and BT-474 (**F**). These were compared to expected values according to CCLE (**E,G**). Note the log_2_ y-axes and the gene names on the x-axes. Complete data is presented in Supplementary Fig. S4.

We applied PV1 to breast cancer cell lines SK-BR-3 and BT-474. Both are well characterised with respect to CNVs, and fully annotated data is available through the Broad Institute’s Cancer Cell Line Encyclopedia (CCLE; https://portals.broadinstitute.org/ccle/data)^42^. Heat map analysis qualitatively revealed large concordance between PV1 detected amplifications and deletions, with those expected in the CCLE data for both cell lines (Fig. 3A). These were confirmed quantitatively through regression analysis, with correlation coefficients of 0.79 and 0.83, demonstrating strong relationships between PV1 and CCLE in SK-BR-3 and BT-474 respectively (Fig. 3B,C). The top ten deletions and amplifications detected by PV1 compared well to those expected according to CCLE in both cell lines (Fig. 3D–G), with notable deletions including *CDKN2A* and *CDKN2B*, and notable amplifications in *ERBB2*, *CDK12* and *MYC*. The data highlight the utility of our panel in detecting larger CNVs, with all detected and expected deletions (copy number <1.5) and amplifications (copy number >2.5) presented in Supplementary Fig. S4. It is also noteworthy that our panel detected 100% of the expected SNVs and indels in both cell lines (data not shown).

### Coriell controls and specificity

As previously stated, the four genomes originally sourced from the Coriell Institute have been extensively characterised by 12 NGS technologies and are used as benchmarks for validation. As per our sensitivity controls above, our panel detected known variants with a sensitivity of >99% in all four samples (Table 3). Precision, which is a measure of reproducibility, was at >97% in all four samples.

**Table 3:** Sensitivity and precision in well characterised genomes, and specificity measure.

Sample	True positive (TP)	False positive (FP)	True negative (TN)	False negative (FN)	Precision**	Sensitivity***	Specificity****
NA12878	602	16	ND	4	0.974	0.993	ND
NA23485	562	13	ND	1	0.977	0.998	ND
NA24149	580	11	ND	1	0.981	0.998	ND
NA24143	552	12	ND	2	0.979	0.996	ND
AIP*	13	0	88,377	0	1.000	1.000	1.000

Abbreviations: ND; Not done. *AIP gene (993 bp) was Sanger sequenced in 89 FPTS samples alongside application of PV1 and PV2. **Precision calculated by TP/(TP + FP). ***Sensitivity calculated by TP/(TP + FN). ****Specificity (*AIP* only) calculated by TN/(TN + FP).

The third essential parameter for validation is specificity, which relies on ‘true negative’ detection, such that a second technology, usually Sanger sequencing, is employed for verification. We did not use Sanger sequencing on our four Coriell samples. However, as stated, Sanger sequencing data was available for *AIP* screening from 89 FPTS patients also screened on our panel. All bases called as wild type in *AIP* using our panel, were shown to be true, providing a specificity value for our panel of 100% (Table 3). This is comparable to current commercially available panels. Given we only tested one gene which encompasses a target region much smaller than the panel in its entirety (~1 kb), one could reasonably speculate that when taking the whole panel into account, that specificity may drop. However, given the extremely low numbers of false positives and false negatives relative to true positives detected across the entire panel (Table 3), we expect that true negatives will be proportionately high, and that any change in specificity would be negligible. Further, our data for true negatives in *AIP* was reproducible across a large number of patients.

### Application of PV2 to FPTS, OSCC and cSCC samples

Following validation, we tested our ability to detect rare variants (≤1% occurrence in control populations) in three cohorts of patients with different tumour types of the head and neck. Pituitary adenomas are common, with 1:1000 patients presenting with clinically significant disease^29,43^. They are classically associated with gross morbidity encompassing the effects of excess hormone production, vision loss, osteoporosis, diabetes, cardiovascular disease, infertility and disfigurement among others, but their low mortality in general has contributed to poor molecular classification^44^; unlike for OSCC and cSCC below, no extensive online databases are available for pituitary tumours.

**Figure 4:**
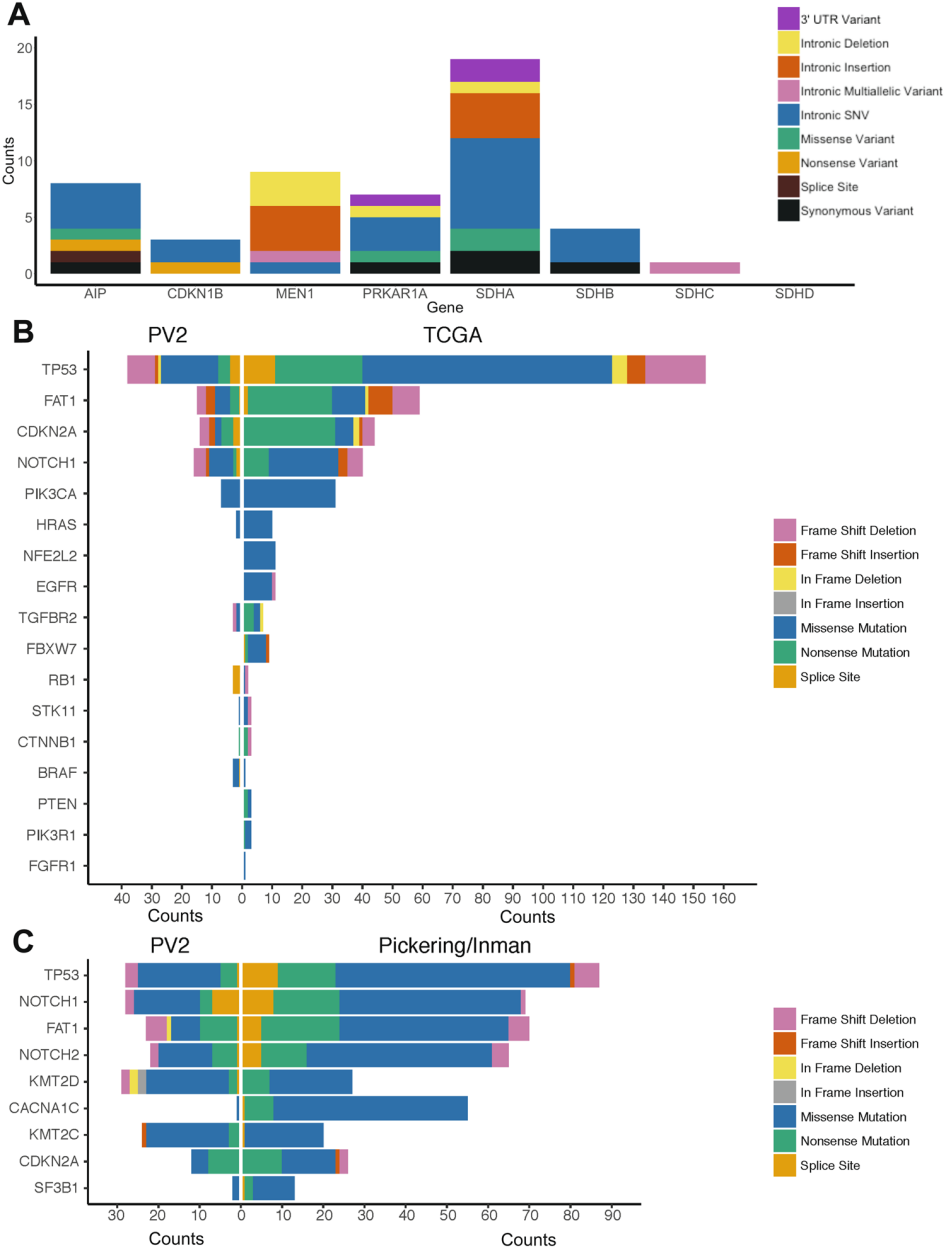
Rare variants are detectable in FPTS, OSCC and cSCC samples, at a similar ratio to online databases. Our validated panel was applied to cohorts of patients with FPTS (n = 18) (**A**), OSCC (n = 39) (**B**), and cSCC (n = 27) (**C**), for the detection of rare (≤1% control population) variations of various categories. Data for OSCC and cSCC was compared to TCGA and Pickering/Inman databases^51,53^, with genes arranged in order of most commonly mutated within those databases. Variants were restricted to high and medium impact. No such database exists for pituitary tumours, and genes were restricted to the 8 described in FPTS. Variants of any impact were included in the data presented herein for FPTS.

Germline variants in eight genes have been associated with FPTS (*AIP*, *MEN1*, *CDKN1B*, *PRKAR1A*, *SDHA*, *SDHB*, *SDHC* and *SDHD*)^29^, and as the occurrence of pathogenic variants is low and we assessed just 18 patients, we included variants of predicted low significance in our data presented herein. SNVs and indels occurring within exons, introns, splice sites and untranslated regions (UTRs) were successfully detected across the genes, except in SDHD (Fig. 4A). Coverage of this gene was a high 479x and hence we postulate that the lack of variants detected was due to the low sample numbers and mutation rarity. Indeed in our previously published data in 44 FPTS patients using the developmental version of this panel, just one predicted benign variant was detected in *SDHD*^29^.

The data presented for our tumour cohorts of OSCC (n = 39; Fig. 4B) and cranio-facial cSCC (n = 24; Fig. 4C), includes variants of high and medium impact only. 8% of worldwide malignancies are mucosal head and neck squamous cell carcinomas, which represent a heterogeneous group of malignancies including OSCC^45^. OSCC is classically associated with alcohol and tobacco abuse in older men^46^. cSCC is associated with UV-induced DNA damage due to sunlight exposure^47^. Its incidence is poorly defined^48^, though like for OSCC, it is increasing. Prognosis is poor for OSCC and also for the <5% of cSCC that is metastatic to regional lymph nodes^49–52^. Genetic stratification for both tumours is improving; for OSCC on The Cancer Genome Atlas (TCGA; a collection of >20,000 extensively-characterised primary tumours spanning 33 cancer types) and for cSCC in the literature^51,53^.

The genes presented in Fig. 4B,C are ordered according to their mutation rate in TCGA and Pickering *et al*.^51^ and Inman *et al*.^53^. Our panel identified mutations in these genes at a similar order of occurrence in our cohorts. The exception is for *CACNA1C* in cSCC which was not targeted by PV2. Consistent with our panel validation data, we were able to detect mutations of various categories, and when taking into account that the TCGA and Pickering/Inman databases used larger cohorts than that used herein, we detected these mutation categories at similar proportions to the databases. Detailed analysis of these three cohorts of data will be presented elsewhere.

### Application of our panel to synthetic ctDNA oligos

Having produced a panel with high sensitivity, precision and specificity, and having demonstrated its application to cancers of the head and neck, we tested the utility of our panel in liquid biopsy; an emerging tool in tumour burden monitoring and early detection of cancer or relapse that aids personalised medicine. The commercially available synthetic oligos used were produced at fragment sizes analogous to ctDNA (167 bp)^54^, suspended in a substrate similar to human plasma, and provided in aliquots of four different VAFs. We achieved a mean depth of coverage of 2,196x across the aliquots, and detected 8/9 and 6/9 mutations at 5.00% and 1.25% VAF respectively (Table 4). We could not detect any expected variants at 0.625% or 0.125% VAF (Table 4).

**Table 4:** Detection of synthetic ctDNA oligos.

Gene/mutation	5.00% VAF (2314x mean coverage)	1.25% VAF (2015x mean coverage)	0.625% VAF (2091x mean coverage)	0.125% VAF (2364x mean coverage)
*BRAF*; p.V600E	Yes	Yes	No	No
*EGFR*; p.T790M	Yes	No	No	No
*EGFR*; p.D770_N771insG	Yes	Yes	No	No
*EGFR*; p.E746_A750del	Yes	Yes	No	No
*PIK3CA*; p.H1047R	Yes	No	No	No
*PIK3CA*; p.N1068fs*4	Yes	Yes	No	No
*NRAS*; p.Q61R	Yes	Yes	No	No
*KRAS*; p.G12D	Yes	No	No	No
*KIT*; p.D816V	No	Yes	No	No

Abbreviation: VAF; Variant Allele Frequency.

Shu *et al*.^54^ employed a panel of similar size to ours and detected tumour specific mutations in 87% of 605 ctDNA samples sourced from various cancer types, with VAF >1% at 300–500x depth. Increasing depth of coverage to ~2000x did not significantly alter variant-detectability^54^. However, in early stage cancer ctDNA can exist at levels around 0.1% VAF, and thus for early detection of cancer initiation or recurrence, previous studies sought to achieve a 10,000x coverage; a target both expensive to achieve and prone to sequencing errors and false positives^54–57^. New technologies have been developed to identify and reduce sequencing errors to enable ultra-deep sequencing of ctDNA^54–59^, but to aid this economically, smaller sequencing panels may need to be developed.

Early data suggest our panel’s applicability for the monitoring of established cancers shedding higher quantities of ctDNA. To detect earlier cancers, or variants in tumours known to shed less ctDNA (eg glioma and renal cell carcinoma)^60^, modifications would need to be made such as reducing the breadth of our target region to common cancer genes or to mutational hotspots, or adopting some of the new, emerging technologies^54–59^.

In conclusion, we have developed a targeted gene panel for application to disparate cancer types that encompasses 451 cancer-associated genes. The novelty of our panel is that compared to many commercially available products, PV2 targets genes in their entirety and not just select exons or mutational hotspots. Additionally, our panel targets a broad range of pituitary tumour-associated genes. We validated our panel by assessing its analytical sensitivity, precision and specificity. This was achieved through application to 47 well-characterised controls, including 4 that are routinely used for benchmarking. Our sensitivity and specificity results were aligned to commercially available panels and early data suggest promise in liquid biopsy, an emerging tool in precision oncology. Finally, we applied our validated panel to cohorts of patients with pituitary tumours and OSCC and cSCC. We were able to detect variants of various categories and for OSCC and cSCC, at similar proportions to those expected, suggesting that our panel would be beneficial to patients with poorly studied cancers of the head and neck, and beyond.

## Supplementary information

Supplementary Information

Supplementary Table S2

Supplementary Table S3

## Data Availability

The patient-derived datasets generated here currently lack consent for genomic data sharing, and as such are not deposited online.

## References

[1] Hoadley KA (2014). Multi-platform analysis of 12 cancer types reveals molecular classification within and across tissues-of-origin. Cell..

[2] Hyman DM, Taylor BS, Baselga J (2017). Implementing genome-driven oncology. Cell..

[3] Druker BJ (2006). IRIS investigators five-year follow-up of patients receiving imatinib for chronic myeloid leaukemia. N. Engl. J. Med..

[4] Bower H (2016). Life expectancy of patients with chronic myeloid leukemia approaches the life expectancy of the general population. J. Clin. Oncol..

[5] Lynch TJ (2004). Activating mutations in the epidermal growth factor receptor underlying responsiveness of non-small-cell lung cancer to gefitinib. N. Engl. J. Med..

[6] Paez JG (2004). EGFR mutations in lung cancer: correlation with clinical response to gefitinib therapy. Science..

[7] Slamon DJ (2001). Use of chemotherapy plus a monoclonal antibody against HER2 for metastatic breast cancer that overexpresses HER2. N. Engl. J. Med..

[8] Freedman AN (2018). Use of next-generation sequencing tests to guide cancer treatment: results from a nationally representative survey of oncologists in the Unite States. JCO. Precis. Oncol..

[9] Miller EM (2017). Development and validation of a targeted next generation DNA sequencing panel outperforming whole exome sequencing for the identification of clinically relevant genetic variants. Oncotarget..

[10] Suwinski P (2019). Advancing personalized medicine through the application of whole exome sequencing and big data analytics. Front. Genet..

[11] Castellanos E (2017). A comprehensive custom panel design for routine hereditary cancer testing: preserving control, improving diagnostics and revealing a complex variation landscape. Sci. Rep..

[12] Lionel AC (2018). Improved diagnostic yield compared with targeted gene sequencing panels suggests a role for whole-genome sequencing as a first-tier genetic test. Genet. Med..

[13] Froyen G (2016). Validation and application of a custom-designed targeted next-generation sequencing panel for the diagnostic mutational profiling of solid tumours. PLoS One..

[14] Matthijs G (2016). Guidelines for diagnostic next-generation sequencing. Eur. J. Hum. Genet..

[15] Jennings LJ (2017). Guidelines for validation of next-generation sequencing-based oncology panels: A joint consensus recommendation of the association for molecular pathology and college of American pathologists. J. Mol. Diagn..

[16] Yohe S (2015). Clinical validation of targeted next-generation sequencing for inherited disorders. Arch. Pathol. Lab. Med..

[17] Celestino-Soper PBS (2017). Validation and utilization of a clinical next-generation sequencing panel for selected cardiovascular disorders. Front. Cardiovasc. Med..

[18] Santani A (2017). Development and validation of targeted next-generation sequencing panels for detection of germline variants in inherited diseases. Arch. Pathol. Lab. Med..

[19] Mu W, Lu HM, Chen J, Li S, Elliott AM (2016). Sanger confirmation is required to achieve optimal sensitivity and specificity in next-generation panel testing. J. Mol. Diagn..

[20] Horak P, Fröhling S, Glimm H (2016). Integrating next-generation sequencing into clinical oncology: strategies, promises and pitfalls. ESMO Open..

[21] Morash M, Mitchell H, Beltran H, Element O, Pathak J (2018). The role of next-generation sequencing in precision medicine: a review of outcomes in oncology. J. Pers. Med..

[22] Frampton GM (2013). Development and validation of a clinical cancer genomic profiling test based on massively parallel DNA sequencing. Nat. Biotechnol..

[23] Bang YJ (2010). Trastuzumab in combination with chemotherapy versus chemotherapy alone for treatment of HER2-positive advanced gastric or gastro-oesophageal junction cancer (ToGA): a phase 3, open-label, randomized controlled trial. Lancet..

[24] Chou A (2013). Clinical and molecular characterization of *HER2* amplified-pancreatic cancer. Genome Med..

[25] Lim SY, Lee JH, Diefenbach RJ, Kefford RF, Rizos H (2018). Liquid biomarkers in melanoma: detection and discovery. Mol. Cancer..

[26] Dawson SJ (2013). Analysis of circulating tumor DNA to monitor breast cancer. N. Engl. J. Med..

[27] Wang J, Bettegowda C (2017). Applications of DNA-based liquid biopsy for central nervous system neoplasms. J. Mol. Diagn..

[28] O’Leary B (2018). Early circulating tumor DNA dynamics and clonal selection with palbociclib and fulvestrant for breast cancer. Nat. Commun..

[29] De Sousa SMC (2017). Germline variants in familial pituitary tumour syndrome genes are common in young patients and families with additional endocrine tumours. Eur. J. Endocrinol..

[30] Zook JM (2016). Extensive sequencing of seven human genomes to characterize benchmark reference materials. Sci. Data..

[31] McCabe MJ (2019). Genomic stratification and liquid biopsy in a rare adrenocortical carcinoma (ACC) case, with dual lung metastases. Cold Spring Harb. Mol. Case. Stud..

[32] Paila U, Chapman BA, Kirchner R, Quinlan AR (2013). GEMINI: integrative exploration of genetic variation and genome annotations. PLoS Comput. Biol..

[33] Gayevskiy V, Roscioli T, Dinger ME, Cowley MJ (2018). Seave: a comprehensive web platform for storing and interrogating human genomic variation. Bioinformatics..

[34] Van der Auwera GA (2013). From FastQ data to high-confidence variant calls: the Genome Analysis Toolkit best practices pipeline. Curr. Protoc. Bioinformatics..

[35] Lai Z (2016). VarDict: a novel and versatile variant caller for next-generation sequencing in cancer research. Nucleic Acids Res..

[36] Talevich E, Shain AH, Botton T, Bastian BC (2016). CNVkit genome-wide copy number detection and visualization from targeted DNA sequencing. PLoS Comput. Biol..

[37] Fowler A (2016). Accurate clinical detection of exon copy number variants in a targeted NGS panel using DECoN. Wellcome Open Res..

[38] Cleary, J. G. *et al*. Comparing variant call files for performance benchmarking of next-generation sequencing variant calling pipelines. *bioRxiv*. 023754 (2015).

[39] Thorvalsdóttir H, Robinson JT, Mesirov JP (2013). Integrative Genomics Viewer (IGV): high-performance genomics data visualization and exploration. Brief. Bioinform..

[40] Weerts MJA (2017). Somatic tumor mutations detected by targeted next generation sequencing in minute amounts of serum-derived cell-free DNA. Sci. Rep..

[41] Halperin RF (2019). Leveraging spatial variation in tumor purity for improved somatic variant calling of archival tumor only samples. Front. Oncol..

[42] Barretina J (2012). The Cancer Cell Line Encyclopedia enables predictive modeling of anticancer drug sensitivity. Nature..

[43] Daly AF (2006). High prevalence of pituitary adenomas: a cross-sectional study in the province of Liege, Belgium. J. Clin. Endocrinol. Metab..

[44] Brue T, Castinetti F (2016). The risks of overlooking the diagnosis of secreting pituitary adenomas. Orphanet J. Rare Dis..

[45] Posner MR (2007). Cisplatin and Fluorouracil alone or with Docetaxel in head and neck cancer. N. Engl. J. Med..

[46] Zygogianni AG (2011). Oral squamous cell cancer: early detection and the role of alcohol and smoking. Head Neck Oncol..

[47] Madan V, Lear JT, Szeimies RM (2010). Non-melanoma skin cancer. Lancet..

[48] Que SKT, Zwald FO, Schmults CD (2018). Cutaneous squamous cell carcinoma: Incidence, risk factors, diagnosis, and staging. J. Am. Acad. Dermatol..

[49] Veness MJ, Palme CE, Morgan GJ (2006). High-risk cutaneous squamous cell carcinoma of the head and neck: results from 266 treated patients with metastatic lymph node disease. Cancer..

[50] Patel SC (2011). Increasing incidence of oral tongue squamous cell carcinoma in young white women, age 18 to 44 years. J. Clin. Oncol..

[51] Pickering CR (2014). Mutational landscape of aggressive cutaneous squamous cell carcinoma. Clin. Cancer Res..

[52] Handler MZ, Goldberg DJ (2018). Cutaneous squamous cell carcinoma of the scalp extending to the skull: a case report and review of the literature. J. Cosmet. Dermatol..

[53] Inman GJ (2018). The genomic landscape of cutaneous SCC reveals drivers and a novel azathioprine associated with mutational signature. Nat. Commun..

[54] Shu Y (2017). Circulating tumour DNA mutation profiling by targeted next generation sequencing provides guidance for personalized treatments in multiple cancer types. Sci. Rep..

[55] Minoche AE, Dohm JC, Himmelbauer H (2011). Evaluation of genomic high-throughput sequencing data generated on Illumina HiSeq and genome analyzer systems. Genome Biol..

[56] Lanman RB (2015). Analytical clinical validation of a digital sequencing panel for quantitative, highly accurate evaluation of cell-free circulating tumour DNA. PLoS One..

[57] Newman AM (2016). Integrated digital error suppression for improved detection of circulating tumor DNA. Nat. Biotechnol..

[58] Newman AM (2014). FACTERA: a practical method for the discovery of genomic rearrangements at breakpoint resolution. Bioinformatics..

[59] Chien R (2017). Comprehensive detection of ctDNA variants at 0.1% allelic frequency using a broad targeted NGS panel for liquid biopsy research. J. Clin. Oncol..

[60] Bettegowda C (2014). Detection of circulating tumor DNA in early- and late-stage human malignancies. Sci. Transl. Med..

