# Supplementary material for: Development and validation of a targeted gene sequencing panel for application to disparate cancers
Source: Sci Rep. 2019 Nov 19;9:17052. doi: 10.1038/s41598-019-52000-3 (PMC6864073; doi:10.1038/s41598-019-52000-3)
Supplement: Supplementary file 1 — Supplementary Information [file 41598_2019_52000_MOESM1_ESM.pdf]

Development and validation of a targeted gene sequencing panel for application to disparate cancers

Mark J McCabe[1,2,3#], Marie-Emilie A Gauthier[1,4,5#], Chia-Ling Chan[1], Tanya J Thompson[2], Sunita MC De Sousa[6,7,8,9], Clare Puttick[1], John P Grady[1], Velimir Gayevskiy[1], Jiang Tao[1], Kevin Ying[1], Arcadi Cipponi[10], Niantao Deng[3,10], Alex Swarbrick[10], Melissa L Thomas[11], kConFab[12,13], Reginald V Lord[11,14], Amber L Johns[10], Maija Kohonen-Corish[10,15,16], Sandra A O'Toole[17,18,19,20], Jonathan Clark[4,21,22], Simon A Mueller[1,4,23], Ruta Gupta[11,22,23], Ann I McCormack[2,3,24], Marcel E Dinger[1,3], Mark J Cowley[1,3,5*]

Supplementary Figure S1. Sensitivity controls. To test panel sensitivity, controls with known single nucleotide variants (SNVs), multiple nucleotide variants (MNVs), small insertions (INS) and deletions (DEL), substitutions (SUBST), duplications (DUP) and complex variants (CV) were obtained from Kathleen Cunningham Foundation Consortium for Research into Familial Breast Cancer (kConFab) or as a commercially available pool of synthetic oligos against a normal genomic background (AcroMetrix). Presented here are pie charts representing the distribution of the different mutation types across the controls. kConFab samples were provided as germline DNA samples from 35 breast cancer patients (7 other samples had previously been omitted), whilst AcroMetrix represented 34 germline mutations, and 521 somatic mutations at 5-15% variant allele frequency (VAF; n=341), and 15-35% VAF (n=180).

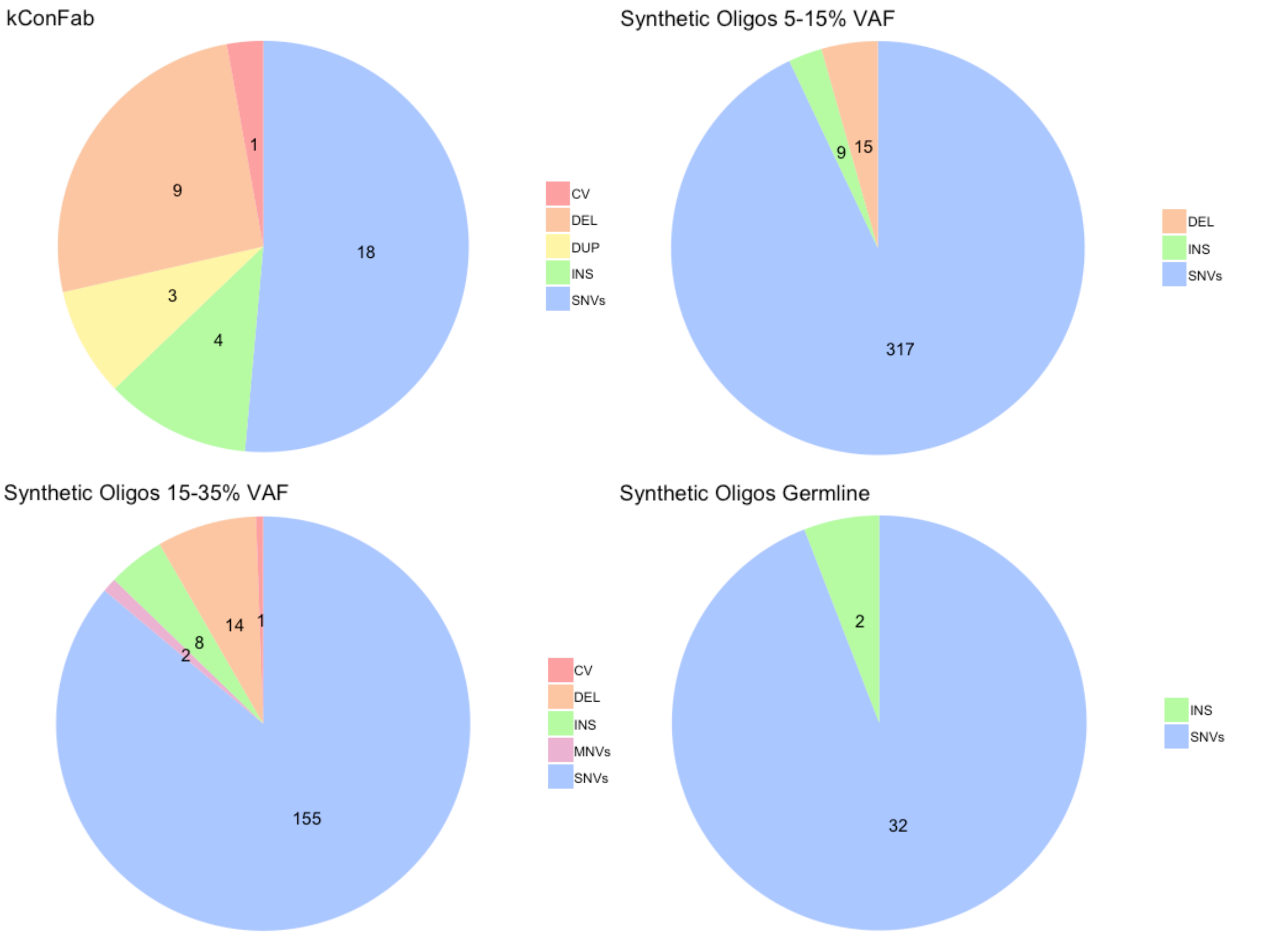
kConFab
1
9
18
3
4
CV
DEL
DUP
INS
SNVs
Synthetic Oligos 5-15% VAF
9
15
317
DEL
INS
SNVs
Synthetic Oligos 15-35% VAF
14
1
8
2
155
CV
DEL
INS
MNVs
SNVs
Synthetic Oligos Germline
2
32
INS
SNVs

Supplementary Table S1. Panel metrics for targeted regions (coding + non-coding)

	PV1			*PV2*			*Fail criteria*
	Mean	***Min***	***Max***	***Mean***	***Min***	***Max***	
Number of reads	21,951,481	11,688,460	46,698,912	22,995,280	9,385,708	54,554,608	
Unique reads	9,263,214	2,799,758	31,500,507	17,417,919	5,529,146	46,378,680	
Mean coverage across target	380	100	1425	395	106	1006	<100x
%Bases > 20x across target	0.95	0.91	1.00	0.99	0.97	1.00	<95%
%Bases > 50x across target	0.93	0.88	0.99	0.96	0.86	0.99	
%Bases > 100x across target	0.89	0.54	0.98	0.88	0.48	0.99	
Read enrichment (%)	81	77	86	68	50	80	
Duplication (%)	59	33	78	26	8	68	

Supplementary Figure S2. Potential annotation errors in three of the kConFab samples. Integrative Genomics Viewer (IGV) screenshots of three variants of discrepant annotation provided by kConFab. All three are in *BRCA1* which is on the antisense strand, hence reverse base nomenclature. (A) Expected heterozygous variant occurring at splice site c.135-1G>T. Detected variant is indicated by the black arrow. (B) An insertion was expected in *BRCA1* at c.3194-3195. This position is indicated by the black arrow in the magnified inset. (C) A 2bp insertion was expected at c.1881_1882 in *BRCA1*. This position is indicated by the black arrow in the magnified inset. Individual reads in opposing directions are shown as purple and orange lines. Mutations/insertions of interest are positioned where actually detected, between the dashed black lines. Note insertions (indicated by a purple I) have been grouped together in the insets for ease of visualisation.

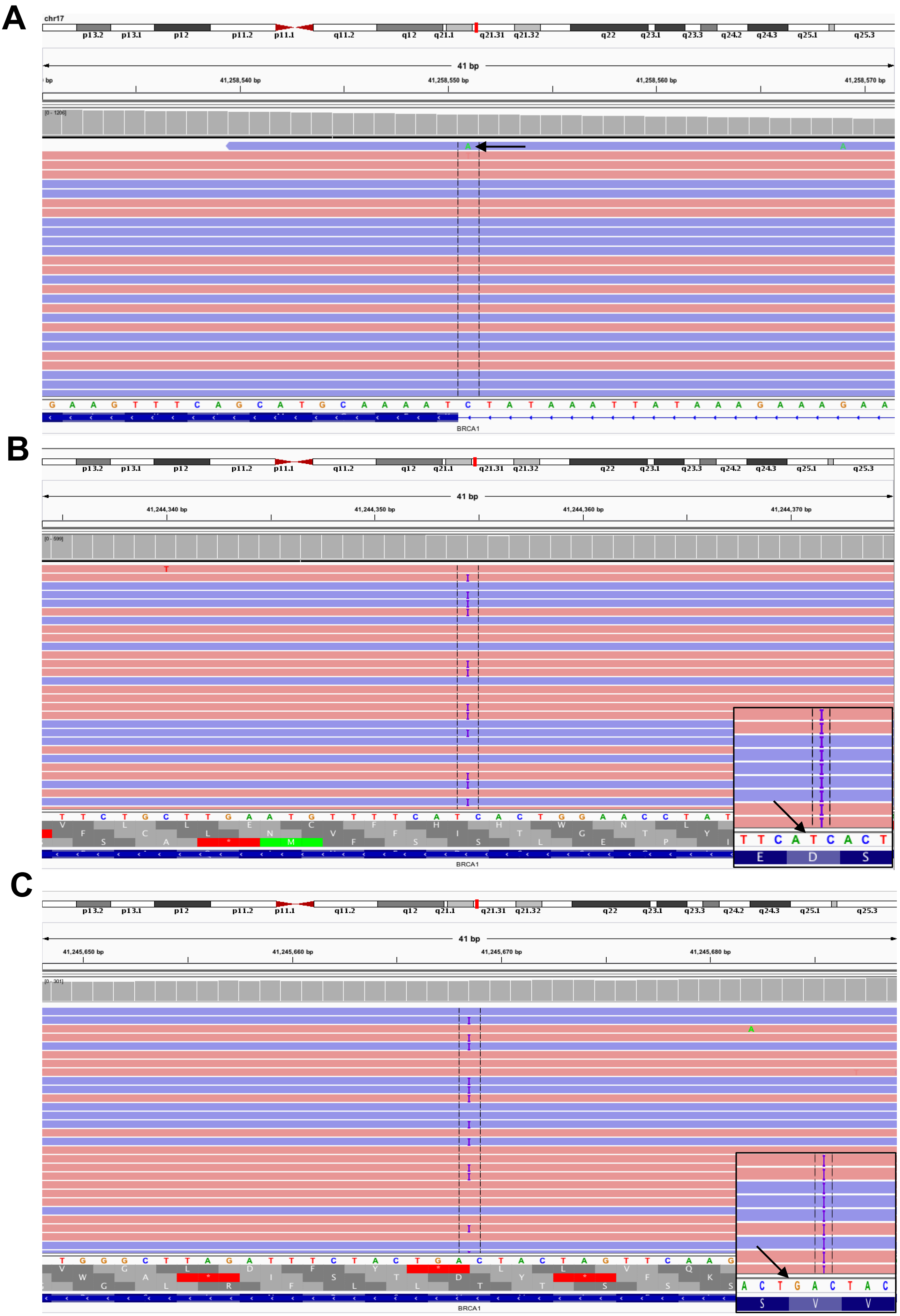

A
chr17
p13.2 p13.1 p12 p11.2 p11.1 q11.2 q12 q21.1 q21.31 q21.32 q22 q23.1 q23.3 q24.2 q24.3 q25.1 q25.3
41 bp
41,258,540 bp
41,258,550 bp
41,258,560 bp
41,258,570 bp
[0 - 1206]
G A A G T T T C A G C A T G C A A A A T C T A T A A A T T A T A A A G A A A G A A
BRCA1
B
41,244,340 bp
41,244,350 bp
41,244,360 bp
41,244,370 bp
[0 - 599]
T T C T G C T T G A A T G T T T T C A T C A C T G G A A C C T A T
T T C A T C A C T
E D S
BRCA1
C
41,245,650 bp
41,245,660 bp
41,245,670 bp
41,245,680 bp
[0 - 301]
T G G G C T T A G A T T T C T A C T G A C T A C T A G T T C A A G
A C T G A C T A C
S V V
BRCA1

Supplementary Figure S3. Improved coverage and missed AcroMetrix variants. Representative Integrative Genomics Viewer (IGV) snapshots for two variants missed in PV1 due to lack of coverage/targeting in (A) kConFab sample *CHEK2* c.441+1G>A and (B) AcroMetrix *PTEN* c.870A>G, p.G293G, that were then readily detectable in the improved PV2 design. Two variants (of 555 targeted), were still missed in the AcroMetrix variant pool including (C) *HNF1A* c.872_873insC, p.G292fs*25 occurring in a highly repetitive region and (D) *PIK3CA* c.1633G>A, p.E545K occurring in a mutational hotspot. Note in C, there are two panels indicating conflicting positioning of the insertion (purple I) either side of the guanine nucleotide. This is the same position as an expected substitution in the background genomic DNA. Individual reads in opposing directions are shown as purple and orange lines. Mutations of interest are positioned between the dashed black lines.

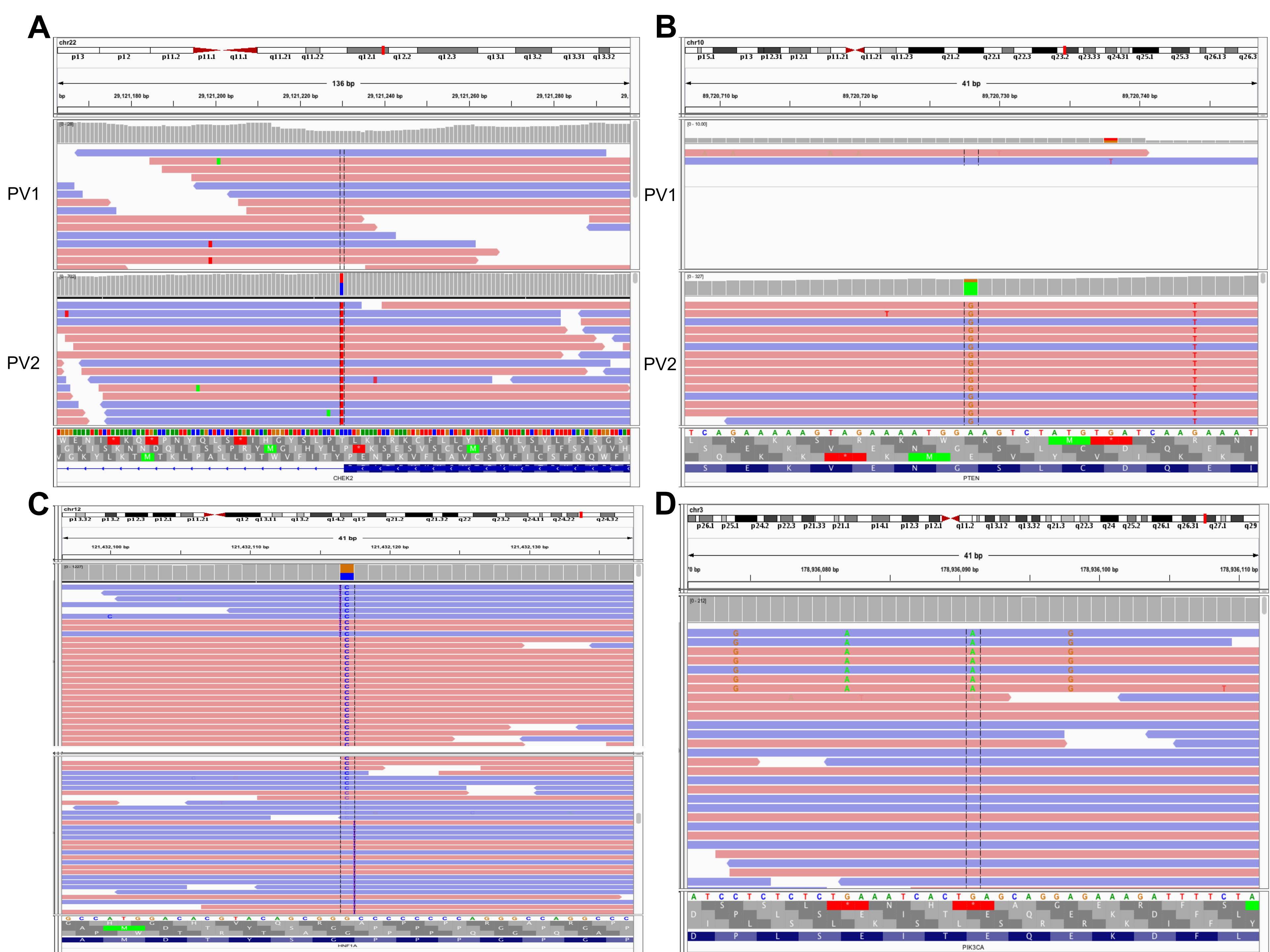
A
B
C
D
PV1
PV2
PV1
PV2
chr22
136 bp
29,121,180 bp
29,121,200 bp
29,121,220 bp
29,121,240 bp
29,121,260 bp
29,121,280 bp
CHEK2
chr10
41 bp
89,720,710 bp
89,720,720 bp
89,720,730 bp
89,720,740 bp
PTEN
chr12
41 bp
121,432,100 bp
121,432,110 bp
121,432,120 bp
121,432,130 bp
HNF1A
chr3
41 bp
178,936,080 bp
178,936,090 bp
178,936,100 bp
178,936,110 bp
PIK3CA

Supplementary Figure S4. Common shared CNVs between PV1 captured DNA and CCLE-reported CNVs. Data presented are an extension upon Fig. 3D-G, and represent all deletions (red) and amplifications (blue) detected by PV1 in the SK-BR-3 and BT-474 breast cancer cell lines in comparison to those reported by the Cancer Cell Line Encyclopedia (CCLE). Deletions were defined as copy numbers of <1.5 and amplifications were defined as copy numbers of >2.5 to allow for errors. Samples which did not fall within these definitions were considered to be diploid and excluded from the plots. Note the log_2 y-axes. Gene names are recorded along the x-axes.

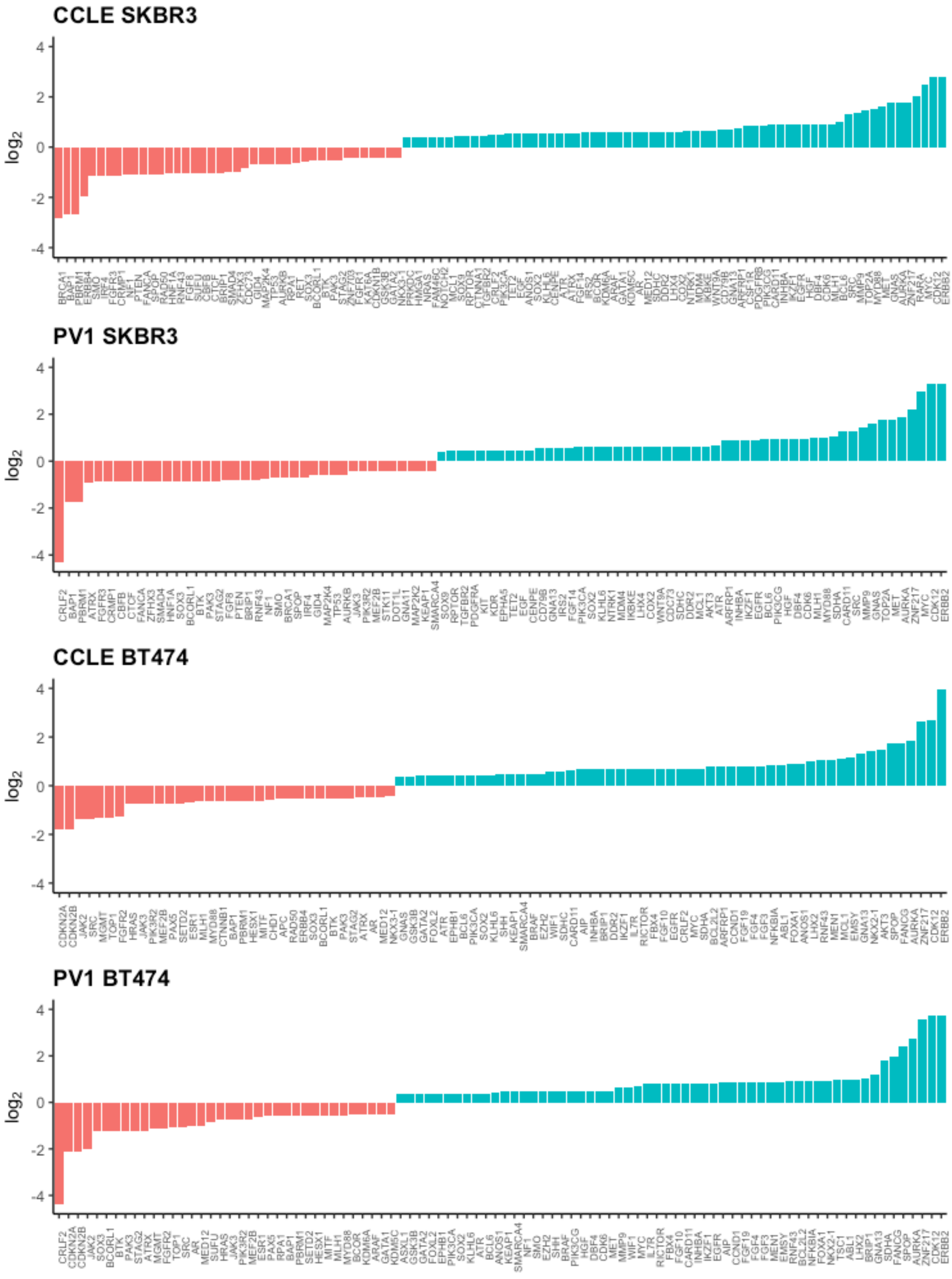
CCLE SKBR3
PV1 SKBR3
CCLE BT474
PV1 BT474
log2
4
2
0
-2
-4
